# Telehealth for the Longitudinal Management of Chronic Conditions: Systematic Review

**DOI:** 10.2196/37100

**Published:** 2022-08-26

**Authors:** Allison A Lewinski, Conor Walsh, Sharron Rushton, Diana Soliman, Scott M Carlson, Matthew W Luedke, David J Halpern, Matthew J Crowley, Ryan J Shaw, Jason A Sharpe, Anastasia-Stefania Alexopoulos, Amir Alishahi Tabriz, Jessica R Dietch, Diya M Uthappa, Soohyun Hwang, Katharine A Ball Ricks, Sarah Cantrell, Andrzej S Kosinski, Belinda Ear, Adelaide M Gordon, Jennifer M Gierisch, John W Williams Jr, Karen M Goldstein

**Affiliations:** 1 Center of Innovation to Accelerate Discovery and Practice Transformation Durham Veterans Affairs Health Care System Durham, NC United States; 2 School of Nursing Duke University Durham, NC United States; 3 Division of General Internal Medicine Department of Medicine Duke University School of Medicine Durham, NC United States; 4 Division of Endocrinology, Diabetes and Metabolism Department of Medicine Duke University School of Medicine Durham, NC United States; 5 Department of Neurology Duke University Medical Center Durham, NC United States; 6 Neurodiagnostic Center Durham Veterans Affairs Medical Center Durham, NC United States; 7 Duke Primary Care Duke University Medical Center Durham, NC United States; 8 Department of Health Outcomes and Behavior Moffitt Cancer Center Tampa, FL United States; 9 School of Psychological Science Oregon State University Corvallis, OR United States; 10 Doctor of Medicine Program Duke University School of Medicine Durham, NC United States; 11 Department of Health Policy and Management Gillings School of Global Public Health University of North Carolina Chapel Hill, NC United States; 12 Cecil G Sheps Center for Health Service Research University of North Carolina Chapel Hill, NC United States; 13 Duke University Medical Center Library Duke University School of Medicine Durham, NC United States; 14 Department of Biostatistics and Bioinformatics Duke University School of Medicine Durham, NC United States; 15 Department of Population Health Sciences Duke University School of Medicine Durham, NC United States; 16 Department of Psychiatry and Behavioral Sciences Duke University School of Medicine Durham, NC United States

**Keywords:** telemedicine, diabetes mellitus, type 2, heart failure, pulmonary disease, chronic obstructive, veterans, delivery of health care, systematic review

## Abstract

**Background:**

Extensive literature support telehealth as a supplement or adjunct to in-person care for the management of chronic conditions such as congestive heart failure (CHF) and type 2 diabetes mellitus (T2DM). Evidence is needed to support the use of telehealth as an equivalent and equitable replacement for in-person care and to assess potential adverse effects.

**Objective:**

We conducted a systematic review to address the following question: among adults, what is the effect of synchronous telehealth (real-time response among individuals via phone or phone and video) compared with in-person care (or compared with phone, if synchronous video care) for chronic management of CHF, chronic obstructive pulmonary disease, and T2DM on key disease-specific clinical outcomes and health care use?

**Methods:**

We followed systematic review methodologies and searched two databases (MEDLINE and Embase). We included randomized or quasi-experimental studies that evaluated the effect of synchronously delivered telehealth for relevant chronic conditions that occurred over ≥2 encounters and in which some or all in-person care was supplanted by care delivered via phone or video. We assessed the bias using the Cochrane Effective Practice and Organization of Care risk of bias (ROB) tool and the certainty of evidence using the Grading of Recommendations Assessment, Development, and Evaluation. We described the findings narratively and did not conduct meta-analysis owing to the small number of studies and the conceptual heterogeneity of the identified interventions.

**Results:**

We identified 8662 studies, and 129 (1.49%) were reviewed at the full-text stage. In total, 3.9% (5/129) of the articles were retained for data extraction, all of which (5/5, 100%) were randomized controlled trials. The CHF study (1/5, 20%) was found to have high ROB and randomized patients (n=210) to receive quarterly automated asynchronous web-based review and follow-up of telemetry data versus synchronous personal follow-up (in-person vs phone-based) for 1 year. A 3-way comparison across study arms found no significant differences in clinical outcomes. Overall, 80% (4/5) of the studies (n=466) evaluated synchronous care for patients with T2DM (ROB was judged to be low for 2, 50% of studies and high for 2, 50% of studies). In total, 20% (1/5) of the studies were adequately powered to assess the difference in glycosylated hemoglobin level between groups; however, no significant difference was found. Intervention design varied greatly from remote monitoring of blood glucose combined with video versus in-person visits to an endocrinology clinic to a brief, 3-week remote intervention to stabilize uncontrolled diabetes. No articles were identified for chronic obstructive pulmonary disease.

**Conclusions:**

This review found few studies with a variety of designs and interventions that used telehealth as a replacement for in-person care. Future research should consider including observational studies and studies on additional highly prevalent chronic diseases.

## Introduction

### Background

As a means to mitigate the risk of viral transmission for both patients and clinicians during the COVID-19 pandemic, many health systems have rapidly converted ≥70% of their outpatient visits to telehealth via phone or video delivery [1-5]. To support this shift, the Centers for Medicare and Medicaid Services in the United States issued an emergency ruling to decrease regulatory requirements for telehealth and created payment parity between in-person care and telehealth delivered via phone or video [6]. Increased telehealth use during the COVID-19 pandemic provided health systems, technology companies, and health care providers experience with telehealth at scale and raised the possibility that telehealth could become a standard option in the postpandemic period. However, concerns remain that care delivered via telehealth is potentially low in quality of care, is difficult to incorporate into workflows, and can exacerbate health disparities [7-10]. Specifically, evidence is needed regarding the efficacy of telehealth as a replacement for in-person care when treating patients.

Extensive literature supports telehealth as a supplement or adjunct to in-person care for the management of chronic conditions [11] such as congestive heart failure (CHF) and type 2 diabetes mellitus (T2DM) [12-14]. These 2 highly prevalent chronic diseases are among the most common and costly conditions affecting approximately 13.4% [15] and 10.5% [16] of all adults in the US, respectively. In addition, CHF and T2DM typically require physical assessment to establish disease status and assess the presence and extent of exacerbations. However, the effects of telehealth as a replacement for in-person health care delivery for CHF, T2DM, and other chronic illnesses remain uncertain [10,17,18] Before the COVID-19 pandemic, many patients with chronic medical conditions, such as CHF, chronic obstructive pulmonary disease (COPD), and T2DM, uniformly received in-person evaluation. During the pandemic, these patients often received telehealth to unknown effect. Although telehealth can increase accessibility to health care by lowering barriers to access [19-21], few studies exist to support the use of telehealth as an equivalent and equitable replacement for in-person care, and the potential adverse effects have not been well defined [18]. Assuming that telehealth can readily replace in-person care may be inappropriate, given the scarcity of evidence examining telehealth applied in this way.

A first step to address the question of equivalence of synchronous (real time) telehealth via phone or video as a replacement for in-person care for chronic diseases is a review focused specifically on evidence from the comparative literature. If there is moderate to strong evidence that telehealth is equivalent to in-person care for patients with chronic conditions, its promise should be developed more fully and incorporated as a standard option for delivering longitudinal care. Early during the COVID-19 pandemic, there was the first complete replacement of telehealth with in-person care [1-5]. However, since then, we have started to see the routine substitution of telehealth for in-person care visits across many specialties and contexts. This substitution (meaning only video) is not usually for all care, but rather can often be a replacement for part of in-person care (some phone visits replaced by video). In addition, currently, there are multiple commercial health care providers who provide only telehealth (Teledoc and CallonDoc). It is within this context that we formulated the questions for this review.

### Objective

We conducted a systematic review to summarize and report the use of telehealth as a replacement or substitute for in-person care in the context of chronic management of CHF, COPD, and T2DM. The questions guiding this review were the following:

1. Question 1a—Among adults, what is the effect of synchronous (real time) telehealth (phone or phone and video) compared with in-person care (or compared with phone, if synchronous video care) for the chronic management of CHF, COPD, and T2DM on key disease-specific clinical outcomes and health care use (eg, hospital admission, hospital readmission, and emergency room visits)?
2. Question 1b—For each disease (CHF, COPD, and T2DM), does this effect differ by race and ethnicity, gender, age, and rural status?
3. Question 2—What are the adverse effects of synchronous telehealth for the chronic management of CHF, COPD, and T2DM as compared with in-person care (or compared with phone, if synchronous video care) on patients?

## Methods

### Overview

This systematic review was conducted as part of a Veterans Health Administration (VHA)–funded report [22] in response to a topic proposed by the VHA Office of Rural Health. For this review, similar to completed previous reviews and to meet the goals of the VHA as a learning health care system [23], (1) the partners from the Office of Rural Health were not involved in conducting the review, but informed topic and question development and provided contextual relevance for the study; (2) the partners from the Office of Rural Health were not involved in approving the final write-up of the report; and (3) a technical expert panel guided the conduct of the review and discussion of the findings. We developed and followed an a priori protocol for this review, and there were no significant deviations after registration (PROSPERO [International Prospective Register of Systematic Reviews] registration number CRD42021239756) [24]. Each step was pilot-tested to train and calibrate the study investigators. We adhered to the PRISMA (Preferred Reporting Items for Systematic Reviews and Meta-Analyses) guidelines [25].

### Analytic Framework

**Figure 1:**
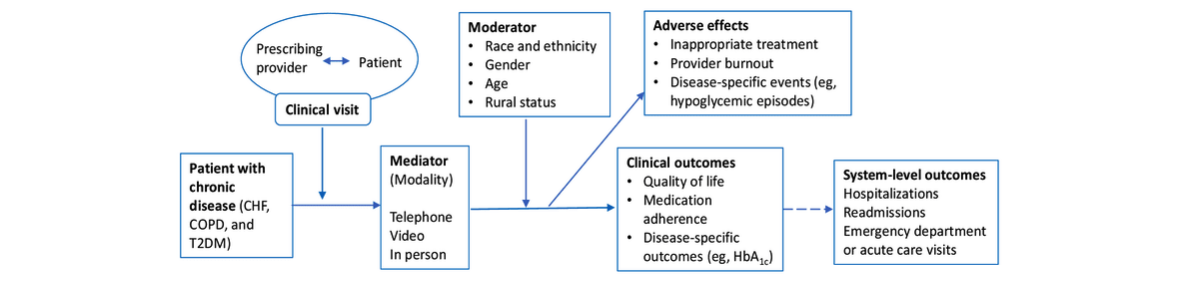
Analytic framework to guide systematic review activities. CHF: congestive heart failure; COPD: chronic obstructive pulmonary disease; HbA_1c_: glycosylated hemoglobin; T2DM: type 2 diabetes mellitus.

We developed an analytic framework [26] (Figure 1) that outlined the population, outcomes, mediation effect of the care visit modality, moderation effect of patient characteristics, and any adverse effects. First, we identified clinical activities (medication management, symptom monitoring, and physical examination) for longitudinal follow-up and the ability to complete them via phone, video, or either of these for CHF, COPD, and T2DM. We determined the relevant aspects that should be abstracted from the eligible literature to obtain critical evidence about conducting a telehealth visit in any clinic setting. Then, with this foundation, we determined that the telehealth modality (eg, telephone, video, and in person) mediates the relationship between the clinical visit and prespecified clinical-level and system-level outcomes. The telehealth interventions matched with our operationalized definition of telehealth and included important contextual elements such as delivery mode (telephone, video, and in person), dose (duration and frequency of contact), and clinical context of care provision. In addition, we specified that care delivered via telehealth should be for clinical activities provided by the prescribing clinician such as for evaluation, diagnosis, or medication prescription and not for the provision of self-management education or other support provided adjunctively by a clinical team member other than the prescribing clinician (eg, nurse care manager), because such interventions have been previously evaluated [11].

### Search Strategy

In collaboration with an expert medical librarian, we conducted a primary literature search from inception to February 7, 2021, in 2 databases (MEDLINE [via Ovid] and Embase [via Elsevier]). We used database-specific subject headings and keywords to search for relevant titles and abstracts (Multimedia Appendix 1). The search strategies were peer-reviewed by a second expert medical librarian before execution using the Peer Review of Electronic Search Strategies Checklist [27]. In addition, we manually searched previous systematic reviews conducted on this or a related topic for potential inclusion.

### Study Selection

Studies identified through our primary search were classified independently by 2 investigators from the study team for relevance to the questions based on the title and abstract from our a priori established eligibility criteria. Study eligibility criteria were organized by population, intervention, comparator, outcome, timing, and setting elements and other criteria such as study design, language, and publication type (Table 1). All studies classified for inclusion by at least one investigator were reviewed at the full-text level. The studies designated for exclusion by one investigator at the title and abstract level were screened by a second investigator. If both investigators agreed on exclusion, the study was excluded. Full-text review included 2 independent reviewers. Conflicts were resolved via discussion. All articles that met the eligibility criteria at the full-text level were included for data abstraction. All results were tracked in an electronic database (EndNote [Clarivate Analytics] for referencing and DistillerSR [Evidence Partners Inc] for data abstraction).

**Table 1:** Study eligibility.

Study characteristics	Inclusion criteria	Exclusion criteria
Population	Adults (aged ≥18 years) with the following chronic conditions: CHF^a^ COPD^b^ T2DM^c^; at least 75% of the sample, if it is a mix of type 1 and type 2 Clinicians or clinics providing telehealth for chronic conditions, if relevant to adverse effects associated with CHF, COPD, and T2DM	Inpatient populations (eg, tele-ICU^d^) Patients receiving care in an ER^e^ or tele–urgent care setting Intervention limited only to the management of complications of CHF, COPD, and T2DM such as stroke, retinopathy, neuropathy, and foot ulcers
Intervention	Synchronous care delivered over ≥2 encounters for the long-term management of relevant chronic conditions in which some or all in-person care is supplanted by telehealth (phone or video) and which is delivered remotely by an independently licensed clinician May include asynchronous telehealth tools (eg, remote monitoring systems), if in both arms	Supplemental nurse care management Telehealth interventions that do not involve synchronous care delivered by a clinician to a patient (eg, 1-way SMS text messages and reminder systems) Telecardiac or telepulmonary rehabilitation
Comparator	In-person care without any telehealth delivery or care delivered via telephone, if compared with video	No comparator
Outcome	Key clinical outcomes (eg, medication adherence, quality of life, and depression) according to condition: CHF—for example, NYHA^f^ functional classification COPD—for example, exercise tolerance and dyspnea T2DM—for example, HbA_1c_^g^ level Clinical use (hospitalization, hospital readmissions, and ER visits or urgent care) Adverse effects (eg, hypoglycemic episodes, inappropriate treatment, and clinician burnout)	Outcomes other than those listed in the inclusion criteria
Timing	No limit	N/A^h^
Setting	Any outpatient setting (general medical or specialty care clinic)	Intervention delivered primarily in hospital inpatient setting (including ER)
Study design	Studies that meet the EPOC^i^ criteria and have prospective data collection, such as the following: Randomized controlled trials Nonrandomized trials Controlled before-after studies Interrupted time series studies or repeated measures studies	Not a clinical study (eg, editorial and letter to an editor) Uncontrolled clinical study Qualitative studies Prospective or retrospective observational studies Clinical guidelines Measurement or validation studies Studies that focus on mixed chronic conditions if results for specified conditions are not reported separately
Countries	OECD^j^	Non-OECD
Publication types	Full publication in a peer-reviewed journal	Letters, editorials, reviews, dissertations, meeting abstracts, and protocols without results

^a^CHF: congestive heart failure.

^b^COPD: chronic obstructive pulmonary disease.

^c^T2DM: type 2 diabetes mellitus.

^d^ICU: intensive care unit.

^e^ER: emergency room.

^f^NYHA: New York Heart Association.

^g^HbA_1c_: glycosylated hemoglobin.

^h^N/A: not applicable.

^i^EPOC: Effective Practice and Organization of Care.

^j^OECD: Organisation for Economic Co-operation and Development includes Australia, Austria, Belgium, Canada, Chile, Czech Republic, Denmark, Estonia, Finland, France, Germany, Greece, Hungary, Iceland, Ireland, Israel, Italy, Japan, Korea, Latvia, Luxembourg, Mexico, the Netherlands, New Zealand, Norway, Poland, Portugal, Slovak Republic, Slovenia, Spain, Sweden, Switzerland, Turkey, the United Kingdom, and the United States.

### Data Extraction and Quality Assessment

Data from published reports were abstracted into a customized DistillerSR database by one reviewer and overread by a second reviewer. Disagreements were resolved by consensus or by obtaining a third reviewer’s opinion. Data elements included descriptors to assess applicability, quality elements, intervention details, and outcomes including adverse events. Key characteristics that were abstracted included participant descriptors (eg, race and ethnicity, gender, age, and rural status), intervention characteristics (eg, clinician type and telehealth modality), comparator, and outcomes (eg, glycosylated hemoglobin [HbA_1c_] level, hospital admission, emergency department visits, and New York Heart Association functional classification). We abstracted all outcomes that were used to evaluate telehealth, but prioritized outcomes identified a priori in collaboration with our partners from the Office of Rural Health and technical expert panel for analysis. Multiple reports from a single study were treated as a single data point, prioritizing results based on the most complete and appropriately analyzed data. When critical data were missing or unclear in the published reports, we requested supplemental data from the study authors. We emailed the authors of 1.6% (2/129) of the studies to obtain additional information and did not receive a reply from any of them. When we did not have sufficient information, we left the field blank.

The investigators who participated in data extraction also completed the quality assessment. Disagreements were resolved by consensus between the 2 investigators or, when needed, by arbitration by a third investigator. For randomized, nonrandomized, and controlled before-after studies, we used the criteria from the Cochrane Effective Practice and Organization of Care (EPOC) risk of bias (ROB) tool [28]. We assigned a summary ROB score (low, unclear, or high) to individual studies. Among the investigators, no ROB disagreements occurred owing to missing results in a synthesis.

The certainty of evidence for each question was assessed using the approach described by Grading of Recommendations Assessment, Development, and Evaluation [29]. We limited the Grading of Recommendations Assessment, Development, and Evaluation ratings to the questions that had at least two included studies. In brief, this approach requires the assessment of four domains: ROB, consistency, directness, and precision. Additional domains to be used when appropriate are coherence, dose-response association, impact of plausible residual confounders, strength of association (magnitude of effect), and publication bias. We considered these domains qualitatively and assigned a summary rating as high, moderate, or low strength of evidence after discussion by a subteam of 5 investigators. In some cases, high, moderate, or low ratings were impossible or imprudent to be provided. In these situations, a grade of *insufficient* was assigned.

### Subgroups of Interest

The research questions guided our subgroup analysis. Prespecified potential effect modifiers included study design characteristics (eg, allocation concealment), disease context (CHF, COPD, or T2DM), and intervention type (eg, telehealth modality). Regarding patient-level characteristics of interest (race and ethnicity, gender, age, and rural status), we looked for analyses conducted within the primary literature that sought to identify effect modifications (eg, subgroup analyses and regression model explanatory variables). Manuscripts included in this review did not specify descriptions of gender or sex. For consistency, we use *gender* throughout the *Results* and *Discussion* sections because the interventions examined are more relevant to self-identity and not specific to one’s biology at birth. However, we realize that this terminology may not reflect patients who would not have self-identified as such.

### Data Synthesis and Analysis

We summarized the primary literature using relevant data abstracted from the eligible studies. Summary tables describe the key characteristics of the primary studies: study design, patient demographics, and details of the intervention and comparator. Owing to conceptual heterogeneity related to the structure, purpose, and delivery of telehealth visits, we did not conduct a meta-analysis, but rather described findings narratively, focusing on identifying patterns in the efficacy and safety of the interventions across conditions and outcome categories.

Continuous outcomes were summarized using the mean patient-level difference (follow-up minus baseline) when the outcome was reported using the same scale. For studies that did not directly report the mean and SD of patient differences, we used the difference in means between the follow-up and baseline. For 20% (1/5) of the studies [30], we computed the SD of the difference based on the reported *P* value for the difference between the 2 arms, assuming the same correlation between follow-up and baseline in each arm. When studies reported only medians and ranges, we translated them into means and SDs [31], and if a study reported only baseline SD, we assumed the same SD at follow-up. Finally, in the absence of other information, we assumed a conservative 0.5 correlation between the follow-up and baseline measures.

### Ad hoc Horizon Scan to Identify Relevant Studies in Progress

Given the limited amount of existing literature we identified that addressed our questions, we sought to assess the pool of ongoing studies that would add relevant findings in the near future. To conduct such a scan of the literature on the horizon, we applied our previously developed search terms to the Cochrane Central Register of Controlled Trials. Notably, we did not apply the same rigor to this process as for our primary search process. At least one reviewer screened the studies identified through the horizon scan at the title and abstract level, and all the included studies were verified by a second reviewer.

## Results

### Overview

**Figure 2:**
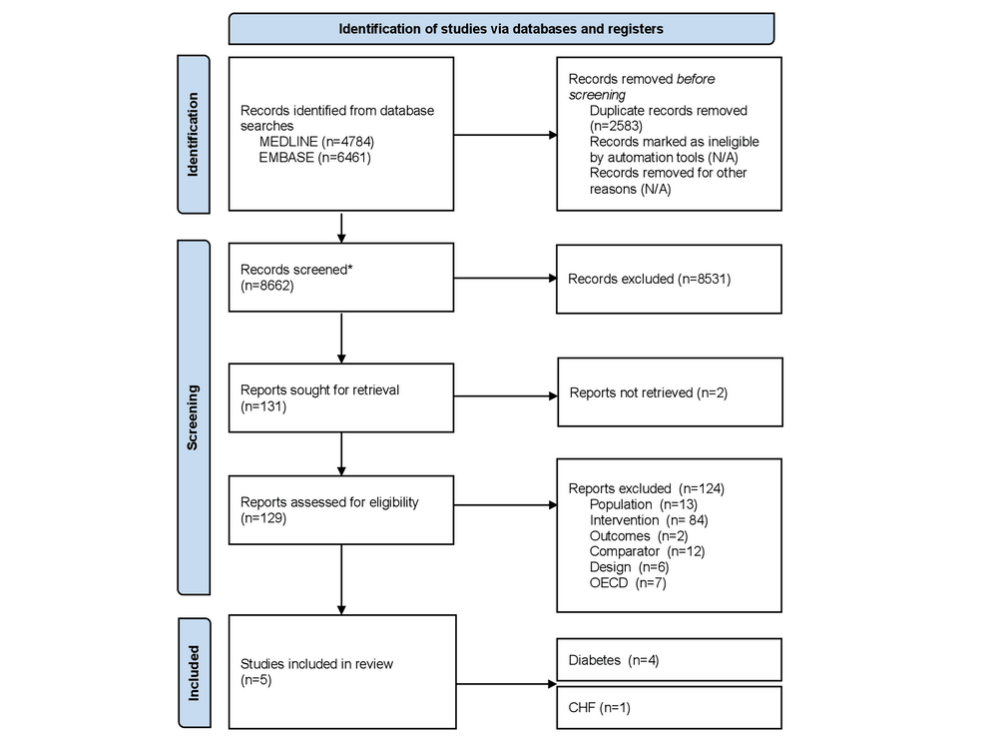
Literature flowchart. *Search results from MEDLINE (4713) and Embase (3949) were combined. CHF: congestive heart failure; N/A: not applicable; OECD: Organisation for Economic Co-operation and Development.

The search identified 11,245 studies from the 2 databases (Figure 2). After deduplication, 77.03% (8662/11,245) of the articles underwent the screening process. In total, 0.06% (5/8662) of the studies met the inclusion criteria. Of those 5 studies, 4 (80%) focused on diabetes and 1 (20%) focused on CHF. The details of the included studies are provided in Table 2. We have provided the details of study characteristics (Multimedia Appendix 2), intervention characteristics (Multimedia Appendix 3), all outcomes reported in the included studies (Multimedia Appendix 4), and excluded studies and the reason for exclusion (Multimedia Appendix 5). Common reasons for excluding studies by intervention included telehealth that supplemented rather than replaced in-person care, telehealth interventions delivered by nonprescribing clinicians, and telehealth delivered asynchronously only. In the following sections, we describe the results by chronic disease (CHF, COPD, and T2DM). The certainty of evidence for the included studies is presented in (Table 3).

**Table 2:** Evidence profile of included studies.

Criteria			Study information
**Region or location (N=5), n (%)**			
	United States		2 (40)
	Europe		2 (40)
	Asia		1 (20)
**Disease in focus (N=5), n (%)**			
	T2DM^a^		4 (80)
	CHF^b^		1 (20)
	COPD^c^		0 (0)
**Patient demographics (N=676)^d^**			
	Age (years), median		58
	**Gender, n (%)**		
		Women	168 (24.9)
		Men	508 (75.1)
	**Race (N=60), n (%)**		
		White^e^	52(87)
		Black^e^	6 (10)
		Hispanic^e^	1 (2)
		Other^e^	1 (2)
**Intervention mode** **(N=5)** **, n (%)**			
	RM^f^ and video		1 (20)
	Video		2 (40)
	RM and telephone		1 (20)
	Telephone		1 (20)
**Comparisons^g^ (N=5), n (%)**			
	RM and in-person care		2 (40)
	Usual in-person care		3 (60)
**Outcomes reported (N=5), n (%)**			
	HbA_1c_^h^ level		4 (80)
	NYHA^i^ functional classification		1 (20)
	Hospitalization		3 (60)
	Emergency department visit		2 (40)
**Risk of bias—objective (N=5), n (%)**			
	High		2 (40)
	Unclear		1 (20)
	Low		2 (40)
**Risk of bias—reported by patient (N=5), n (%)**			
	High		2 (40)
	Unclear		1 (20)
	Low		1 (20)
	N/A^j^		1 (20)

^a^T2DM: type 2 diabetes mellitus.

^b^CHF: congestive heart failure.

^c^COPD: chronic obstructive pulmonary disease.

^d^Of the 5 studies, 1 (20%) study [32] reported 50% (338/676) of the participants.

^e^In total, 80% (4/5) of the studies did not report this information.

^f^RM: remote monitoring.

^g^For this criterion, ≥1 category is possible per study.

^h^HbA_1c_: glycosylated hemoglobin.

^i^NYHA: New York Heart Association.

^j^N/A: not applicable.

**Table 3:** Certainty of evidence for included studies of CHF^a^ and T2DM^b^.

Outcomes		Studies (randomized controlled trials; N=5), n (%)	Patients (N=676), n (%)	Range of effects	*P* value	Certainty of evidence (rationale)
**T2DM**						
	HbA_1c_^c^ level	4 (80)	339 (50.1)	Mean difference of −0.15% to −1.30% in the HbA_1c_ level between the intervention and comparator arms	N/A^d^	Very low certainty that telehealth has an effect on HbA_1__c_ level (rated down for serious risk of bias, indirectness, and imprecision)
	Hospital admission	2 (40)	285 (42.2)	In total, 0 to 3 admissions in the intervention arm and 0 to 7 admissions in the comparator arm	N/A	Very low certainty that telehealth has an effect on hospital admissions (rated down for serious risk of bias, indirectness, and imprecision)
	Emergency department visits	2 (40)	285 (42.2)	In total, 0 emergency department visits in the intervention arm and 0 to 1 visit in the comparator arm	N/A	Very low certainty that telehealth has an effect on emergency department attendance (rated down for serious risk of bias, indirectness, and imprecision)
**CHF**						
	NYHA^e^ functional classification	1 (20)	219 (32.4)	Between-group difference	.97	Very low certainty that telehealth has an effect on NYHA functional classification (rated down for serious risk of bias, inconsistency, indirectness, and imprecision)
	Hospital admission	1 (20)	219 (32.4)	RM^f^ (9.8%), RM and phone (11.3%), and in-person visit (12.7%)	.85	Very low certainty that telehealth has an effect on hospital admission (rated down for serious risk of bias, inconsistency, indirectness, and imprecision)

^a^CHF: congestive heart failure.

^b^T2DM: type 2 diabetes mellitus.

^c^HbA_1c_: glycosylated hemoglobin.

^d^N/A: not applicable.

^e^NYHA: New York Heart Association.

^f^RM: remote monitoring.

### Question 1a (Effect of Telehealth) and 1b (Differences by Special Population)

#### Findings for CHF

##### Question 1a: Effect of Telehealth

###### Overview

We identified only 20% (1/5) of studies that met the inclusion criteria for synchronous telehealth for chronic CHF management [33] and found it to have high ROB. The study was conducted in Germany, enrolled 210 patients, and had a duration of 12 months. The study incorporated phone-based appointments and follow-up in patients with CHF with recent placement of an implanted cardioverter defibrillator or cardiac resynchronization therapy defibrillator. Patients were randomized to receive asynchronous web-based automated review and follow-up of telemetry data alone every 3 months (n=102) or personal physician contact every 3 months in addition to remote monitoring. The personal contact group was further randomized to personal contact via telephone calls (n=53) or personal contact via in-person visits (n=55). In this study [33], the primary outcome was the proportion of patients with worse Packer Heart Failure Clinical Composite Response scores at 13 months compared with scores at 1 month after device placement. The Packer Heart Failure Clinical Composite Response score provides stepwise assessment and incorporates CHF death or hospitalization, change in New York Heart Association class, and self-assessed health status. The secondary outcomes in this study were all-cause mortality, CHF-related hospitalizations, arrhythmias, and changes in reported quality of life. We present the detailed results by outcome: (1) Packer Heart Failure Clinical Composite Response Score, (2) hospitalizations, (3) emergency department visits, and (4) number of contacts and use.

###### Packer Heart Failure Clinical Composite Response Score

The primary outcome of the study by Hansen et al [33] showed no significant differences in Packer scores in a 3-way comparison between the telemetry arm compared with the personal contact subgroups (remote monitoring and phone call vs remote monitoring and in-person visit; *P*=.97).

###### Hospitalizations

The authors found no significant differences between the subgroups in any of the outcomes that were measured. Outcomes between study arms included the following: mortality (*P*=.65), CHF-related hospitalization (*P*=.85), detection of supraventricular tachycardia (*P*=.22), detection of ventricular tachycardia (*P*=.75), and reported change in quality of life (*P*=.72).

###### Emergency Department Visits

The CHF study that was included did not report on emergency department visits [33].

###### Number of Contacts and Use

The CHF study compared the number of unscheduled follow-up visits conducted either via phone or in person among the telemetry only group, telemetry and phone visit group, and telemetry and in-person visit group [33]. In total, there were 219 unscheduled follow-ups among the 3 groups, involving 83 patients during the course of the study. However, there were no significant differences in the unscheduled follow-up rates among the 3 groups (*P*=.29).

##### Question 1b: Differences by Special Population

The 20% (1/5) studies that met the inclusion criteria [33] described the age (overall mean 63.8 years, SD was not reported by authors) and gender of their patient population (84.3% were men); however, details regarding race and ethnicity and rural status were not reported. Furthermore, the authors did not perform any subgroup analyses to examine the effect of age or gender on outcomes.

#### Findings for COPD

No studies that addressed the use of telehealth as a substitute for in-person chronic management of COPD were identified.

#### Findings for T2DM

##### Question 1a: Effect of Telehealth

###### Overview

We identified 80% (4/5) of studies—all of which were randomized controlled trials [30,32,34,35]—that evaluated the provision of synchronous telehealth compared with in-person care for the chronic management of T2DM. Of the 4 studies, 2 (50%) studies were conducted in the United States [30,35], 1 (25%) in South Korea [32], and 1 (25%) in Denmark [34]. Overall, 25% (1/4) of the studies were conducted with patients in the military [35]. Intervention duration varied across studies, from <8 weeks to 52 weeks. Intervention approach varied across all the studies (4/4, 100%) regarding duration and mode of incorporating telehealth into chronic diabetes management. Of the 4 studies, 3 (75%) studies included ≤60 patients [30,34,35] and 1 (25%) study included 338 patients [32]. Of the 4 studies, 3 (75%) studies used technology that facilitated synchronous bidirectional communication between the patient and clinician [32,34,35], and 1 (25%) study relied on telephone and email [30]. In total, 50% (2/4) of the studies included remote monitoring in addition to synchronous telehealth [32,35]. We present the detailed results by outcome: (1) HbA_1c_ level, (2) hospitalizations, (3) emergency department visits, and (4) number of contacts and use.

###### Change in Reduction of HbA_1c_ Level

**Figure 3:**
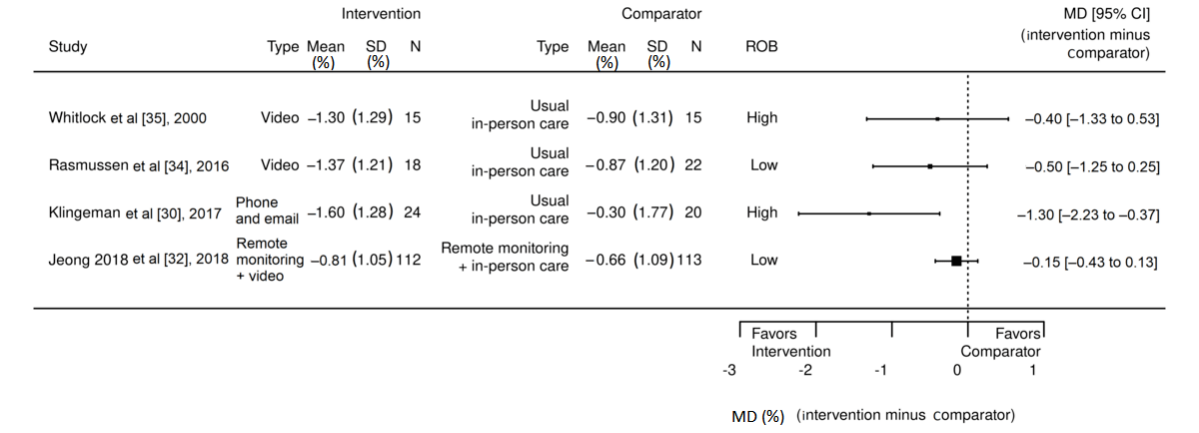
Change in glycosylated hemoglobin levels between intervention and comparator arms across type 2 diabetes mellitus studies. MD: mean difference; ROB: risk of bias [30,32,34,35].

All the studies (4/4, 100%) compared the change in reduction of HbA_1c_ level from baseline to the end of the study between synchronous telehealth and in-person study arms (Figure 3) [30,32,34,35]. The first study, by Jeong et al [32], was a 24-week 3-arm trial that compared usual care, telemonitoring (remote monitoring with automated clinical decision support with in-person endocrine follow-up appointments), and telemedicine (remote monitoring with automated clinical decision support with video-based endocrine follow-up appointments). Notably, that study was the largest study included and was rated as having low ROB. They enrolled 338 patients, with a baseline mean age of 53 years (SD was not reported by authors). No statistically significant difference was seen at baseline for HbA_1c_ level across groups: usual care (mean 8.39%, SD 1.10%), telemonitoring (mean 8.21%, SD 0.93%), and telemedicine (mean 8.39%, SD 1.10%). Statistically significant difference was seen for within-group reduction in HbA_1c_ level from baseline to 24 weeks for all groups, ranging from −0.66% to −0.81% (*P*<.001). No statistically significant difference was noted for the extent of HbA_1c_ reduction across groups: usual care versus telemonitoring groups (*P*=.61), usual care versus telemedicine groups (*P*=.16), and telemonitoring versus telemedicine groups (*P*=.34).

The second study, led by Klingeman et al [30], was a 52-week, 2-arm trial consisting of usual endocrine care versus an experimental endocrine clinic group that enrolled 60 patients with T2DM. The setting for the study was an endocrinology clinic at an academic medical center, where patient care was provided by endocrinologists. Patients who were not in the experimental arm received the usual care provided by the clinic’s endocrinologists. The specialty clinic model in the experimental group included an endocrinologist and nurse educator who focused on patients with advanced diabetes; contact with the patients in this arm was designed to be variable and patient-specific. Preplanned contacts (via email and phone) were determined at baseline and amended over time, and ad hoc in-person visits occurred if clinically required. Contact was individually tailored based on each patient’s outcomes, adverse reactions, and changes in the disease state. The control arm received the usual endocrine care, which included the ability for the patients to contact (via email and phone) clinicians as needed. HbA_1c_ levels were compared between groups at baseline for usual care (mean 8.9%, SD 0.8%) versus specialty clinic model (mean 9.5%, SD 0.9%). In addition, high proportion of patients who were White were enrolled in the intervention group (96.6%) compared with the usual care group (76.8%). Analysis of data at 52 weeks found great reduction in HbA_1c_ level in the specialty clinic model (−1.7%; from 9.6% to 7.9%) as compared with the usual endocrine care (0.3%; from 8.9% to 8.6%), with *P*=.004. Notably, sensitivity analysis was conducted that dropped data from a patient who was an outlier in the usual care group, with worsened HbA_1c_ values (from 8.3% to 13.5%); however, this did not change the results.

The third study, by Rasmussen et al [34], was a 2-arm trial comparing 3 weeks of brief standard in-person endocrine care versus telemedicine (video-based endocrine care) to stabilize patients with poorly controlled T2DM. They enrolled 40 patients with baseline HbA_1c_ level of 8.1% (range 6.1%-10.7%) in standard care group and 9% (range 7.6%-12%) in the telemedicine group. At 6 months, the HbA_1c_ level ranged from 8.1% to 7.2% in the standard care group and from 9.1% to 7.7% in the telemedicine group. The patients in the telemedicine arm experienced a larger decrease in HbA_1c_ level (14.6%) than those in the standard care arm (10.6%), which was statistically significant (*P*=.02). Notably, although this study framed its hypothesis as “the treatment by telemedicine at home was similar to standard care,” the analysis methods did not use noninferiority analytic approaches.

The fourth study, by Whitlock et al [35], which tested usual care and telemonitoring visits with a case manager and physician, enrolled 28 patients in a 36-week 2-arm trial consisting of a standard of care control versus experimental telemonitoring group. In this study, both groups were referred for multidisciplinary diabetic education classes. The experimental group received weekly telemonitoring via video from a case manager and, then, monthly telemonitoring via video from study physicians. Patients in the standard of care group received routine in-person care from their primary care clinician. Statistically significant within-group difference (*P*=.05) was noted for the experimental telemonitoring arm, from baseline HbA_1c_ level of 9.5 (range 8.1-12.6) to week-36 HbA_1c_ level of 8.2 (range 5.7-10.2). For the comparator, the mean baseline HbA_1c_ level was 9.5 (range 8.1-11.9) and week-36 HbA_1c_ level was 8.6 (range 7.1-11.9), which was not statistically significant.

###### Hospitalizations

In total, 50% (2/4) of the studies examined hospitalizations [30,32]. In the study by Jeong et al [32], only 1 patient in the telemonitoring arm experienced a diabetes complication–related hospitalization, and none of the patients in the control or telemedicine arms experienced diabetes-related hospitalizations. In the second study, by Klingeman et al [30], 10% (3/30) of the patients in the experimental arm and 23% (7/30) of the patients in the control arm experienced diabetes-related hospital admission.

###### Emergency Department Visits

Overall, 50% (2/4) of the studies examined emergency department visits [30,32]. In the first study, by Jeong et al [32], across the 3 study arms, none of the patients experienced diabetes-related visits to the emergency department out of the 338 patients enrolled in the study. In the second study, by Klingeman et al [30], none of the patients in the experimental arm and 1 patient in the control arm experienced a T2DM-related emergency department visit.

###### Number of Contacts and Use

In total, 75% (3/4) of the studies reported collecting data on number of contacts and use [30,34,35] among patients receiving in-person or telehealth. The study by Klingeman et al [30] reported on (1) diabetes education referrals, (2) diabetes-related visits, (3) use of modality, and (4) number of interactions and HbA_1c_ level. Klingeman et al [30] designed the experimental arm for variable frequency of contact using a specialty clinic model. Preplanned contacts (via email, phone call, or visit) were determined at baseline and amended over time; contact was tailored based on each patient’s outcomes, adverse reactions, and changes in the disease state; and the control arm received usual endocrine care. Klingeman et al [30] reported that when diabetes education visits were combined with clinician’s diabetes-related visits in the endocrinology clinic, the experimental group had fewer overall visits than the control group. Specifically, the experimental group had 1.5 (SD 0.7) visits and the control group had 3.6 (SD 4) visits over 12 months (*P*<.001). However, the experimental group had significantly more email contacts (mean 11.1, SD 6.4) than the control group with (mean 1.8, SD 3.5; *P*<.001; *note that email communication was a focus in the experimental group*).

The study by Rasmussen et al [34], which tested standard care and video consultation for home treatment of T2DM, reported on (1) number of visits and missed visits and (2) consultation time. The telemedicine group had an average of 4.1 visits, with no missed visits; however, the usual care group had an average of 3.8 visits, with 13% missed visits. Regarding consultation time, the telemedicine group had an average of 18 minutes and the usual care group had an average of 23 minutes. The study by Whitlock et al [35] reported no results on the number of contacts and use, despite describing collecting the number of clinic visits before and during the study in their Methods section.

##### Question 1b: Differences by Special Population

Only 25% (1/4) of the included studies reported on subgroup analysis [32] by patient characteristics. Jeong et al [32] analyzed two subgroups of a priori interest: gender and age. No statistically significant difference in reduction of HbA_1c_ level was found for men (mean −0.76%, SD 1.11% for telemonitoring vs mean −0.89%, SD 1.12% for telemedicine; *P*=.88) or women (mean −0.46%, SD 1.05% vs mean −0.63%, SD 0.87%; *P*=.16). No statistically significant difference in reduction of HbA_1c_ level was seen among people aged <55 years (mean −0.63%, SD 1.26% for telemonitoring vs mean −0.87%, SD 1.15% for telemedicine; *P*=.21) or among those aged ≥55 years (mean −0.68%, SD 0.88% for telemonitoring vs mean −0.73%, SD 0.93% for telemedicine; *P*=.83). Moreover, Jeong et al [32] reported on additional subgroups of potential interest. Users with high compliance (defined as users with >90% of number of records or data transmitted compared with recommended number of records) had no difference in reduction of HbA_1c_ level when compared with those with low compliance levels across the study arms of interest (mean −0.93%, SD 0.99% for telemonitoring vs mean −1.08%, SD 0.96% for telemedicine; *P*=.47). Similarly, there was no significant difference in the reduction of HbA_1c_ level between patients who had a high school education or less in the telemonitoring (mean −0.65%, SD 0.93%) and telemedicine (mean −0.94%, SD 1.1%) arms (*P*=.26).

### Question 2: Adverse Events

The 20% (1/5) of the studies of CHF, by Hansen et al [33], did not report on adverse events. The 40% (2/5) of studies of T2DM reported adverse events [30,32]. Jeong et al [32] described four groups of adverse events: (1) general events, (2) diabetes-related events, (3) serious events, and (4) biochemical events. Adverse events were noted in the control (n=33 or 29.20%, in-person appointments at 8, 16, and 24 weeks), telemonitoring (n=30 or 26.55%, in-person appointments at 8, 16, and 24 weeks, with remote monitoring of blood glucose data), and telemedicine (n=23 or 20.54%, video visits at 8 and 16 weeks, in-person visits at 24 weeks) arms. Diabetes-related events were noted in the control (n=7 or 6.19%), telemonitoring (n=7 or 6.19%), and telemedicine (n=3 or 2.68%) arms. Serious reported adverse events were noted in the control (n=2 or 1.7%), telemonitoring (n=2 or 1.70%), and telemedicine (n=1 or 0.90%) arms, and it included angina pectoris, rotator cuff syndrome, malignant hepatic neoplasm, skin ulcer, and hematuria [32]. Biochemical parameters for serum alanine aminotransferase (ALT), aspartate aminotransferase, and creatinine levels were measured, and samples were obtained at baseline and 24 weeks [32]. Comparing the relative percentage of patients with worsened laboratory values, ALT was the only parameter that showed significant worsening between the telemedicine and telemonitoring groups. Specifically, none of the participants in the telemonitoring arm and 7 participants in the telemedicine arm (6.7%; *P*=.01) experienced worsening of ALT values. Klingeman et al [30] described two types of adverse events: (1) severe hypoglycemia and (2) foot ulcers. Severe hypoglycemia was noted in the experimental (n=1 or 3.3%) arm, but not in the control (n=0 or 0%) arm. Foot ulcer was noted in the experimental (n=1 or 3.3%) and control (n=3 or 10%) arms.

### Quality of Evidence for Included Studies

**Figure 4:**
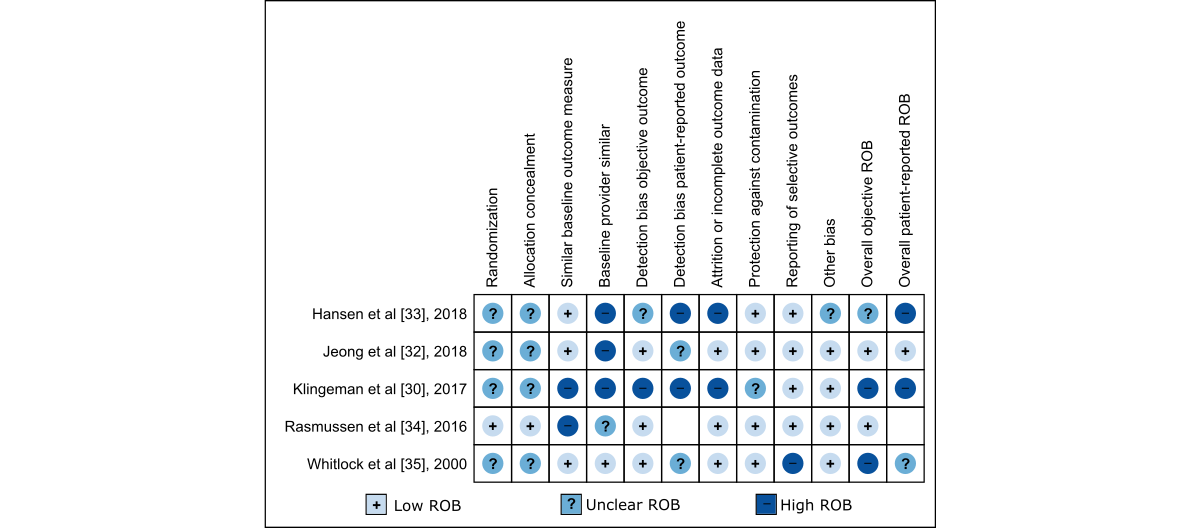
Risk of bias (ROB) assessment for included studies in congestive heart failure and type 2 diabetes mellitus [30,32-35].

**Figure 5:**
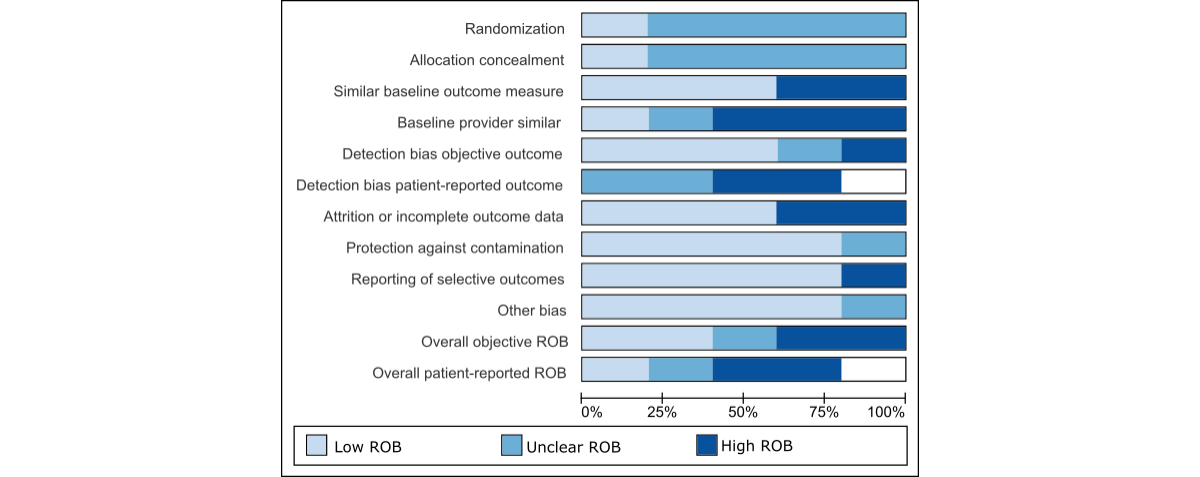
Risk of bias (ROB) assessment across included studies on congestive heart failure and type 2 diabetes mellitus (N=5).

The 20% (1/5) of the studies of CHF [33] that met our inclusion criteria was rated as having high ROB owing to low numbers of patients enrolled, unclear method for patient randomization, and poor description of both patient dropout and how primary outcomes were assessed. Among the 80% (4/5) of randomized T2DM studies, the ROB (Figure 4) for patient-reported outcomes was judged to be low for 1 (25%) study, unclear for 1 (25%) study, and high for 1 (25%) study and 1 (25%) study did not report this type of outcome [30,32,34,35]. For objective outcomes, ROB was judged to be low for 50% (2/4) of the studies [32,34] and high for 50% (2/4) of the studies [30,35]. Patterns that led to judgments of low ROB (Figure 5) included (1) noting randomization of study participants, (2) collecting objective outcome data, and (3) generally limited expected impact of bias from patient knowledge of the treatment arm. Patterns that led to high ROB included (1) missing or unclear data on randomization methods, data collection, and analysis; (2) unblinded treatment arm; (3) absence of predetermined intervention assessment patterns in the protocol; (4) unclear primary outcomes; and (5) missing or unclear reporting of patient-reported outcomes.

### Ad hoc Horizon Scan to Identify Relevant Studies in Progress

This search identified 1787 unique studies. We found only 0.17% (3/1787) of studies [36-38] in our horizon scan that reported on studies without published results (Multimedia Appendix 6) that may potentially meet the inclusion criteria of our systematic review. All of these studies (3/3, 100%) are randomized controlled trials that were designed before the COVID-19 pandemic. Of the 3 studies, 2 (67%) studies focus on T2DM [36,37], whereas the remaining 1 (33%) study is on CHF [38]. Although the noninferiority study [37] will not meet our inclusion criteria as it is conducted in Brazil (a non–Organisation for Economic Co-operation and Development [OECD] country) and the findings may not be applicable to the US population or setting, we mention it here given the low number of studies that otherwise met our inclusion criteria. The other T2DM study [36] is specifically focused on reducing emergency diabetes care for older (aged >50 years) African Americans. The CHF study by Komkov et al [38] has very limited detail available. Although using these registries to identify trials has limitations and there are likely other relevant studies, it appears that there are few trial-based studies currently in the pipeline to inform our questions in this review.

## Discussion

This review aimed to summarize and report the use of telehealth as a replacement or substitute for all or a portion of in-person care in the context of chronic management of CHF, COPD, and T2DM.

### Principal Findings

We found scant evidence examining chronic disease management delivered through synchronous telehealth compared with in-person delivery for T2DM (4/5, 80%), COPD (0/5, 0%), and CHF (1/5, 20%). This suggests that there is little evidence to help guide practice on when to use telehealth instead of traditional in-person visits while managing these chronic diseases. Our review sought to include studies that used telehealth to replace all or part of in-person care. In other words, some specific in-person visits in the intervention arm were replaced by telehealth visits, whereas the comparator arm maintained all visits as in-person. Note that we consider this to be different from using telehealth as a supplement or add-on to the usual in-person care. However, we did not find any studies that only partially substituted in-person visits. We did not attempt to include studies that used telehealth as an add-on to existing in-person care, as there are already existing high-quality, peer-reviewed publications on this question [11-13]. However, despite the paucity of evidence, telehealth modalities such as video or telephone have increasingly been used to replace in-person clinic visits for managing chronic conditions, particularly during the COVID-19 pandemic [1-5]. Understanding the benefits and risks associated with shifting in-person care to telehealth is critical in shaping how health systems deliver care going forward. Although in-person visits have since increased as more has become known about COVID-19 transmission and prevention practices, telehealth continues to play a much larger role in outpatient care than before the pandemic [39,40].

### Comparison With Previous Studies

Evidence indicates that telehealth can be used effectively as an adjunctive or supplemental approach to in-person care. A recent review by Albritton et al [18] examined the impact of video teleconferencing visits on prevention and management of chronic illness. Results from that review indicated that video teleconferencing resulted in similar clinical effectiveness as in-person care for certain diseases [18]. The results from our review differ in indicating clinical effectiveness of telehealth from those of Albritton et al [18] owing to several differences in the review type (systematic vs rapid review), date limitations, search strategies, databases searched, and operationalization of telehealth. Our approach to identify relevant telehealth papers was broad and more comprehensive, which resulted in a large number of articles to review. Of the 7 papers included in the review by Albritton et al [18], only 1 (14%) was not captured in our search. Additional previous reviews have examined various ways of using telehealth modalities in the context of these conditions of interest, but none of them have focused on *replacing* in-person care with telehealth visits [11]. Although we found only 20% (1/5) of the studies on telehealth for chronic management of heart failure as a substitute for in-person care, previous reviews report mixed results for the impact of other supplemental types of telehealth on heart failure outcomes [41-43]. Several recent analyses on the impact of telehealth in T2DM indicated that health outcomes did not worsen because of switching to telehealth compared with those in-person clinic care [14,40,44,45]. However, there is evidence that telehealth as an *adjunctive* strategy to typical in-person care can be associated with a decrease in HbA_1c_ level in patients with both type 1 and type 2 diabetes [46-49].

### Importance of Context in Telehealth Implementation

The successful incorporation of telehealth into health care delivery relies upon the fit between the telehealth modality, care delivery context, and disease management approaches [9,19,50]. Presumably, not all areas of health care delivery lend themselves equally well to telehealth, but management of certain chronic diseases (CHF, COPD, and T2DM) may provide good opportunity to replace routine in-person care with telehealth. In our review, we sought to address a critical evidence gap by examining the comparative literature on telehealth as a replacement for in-person care in chronic disease management. Interestingly, our findings came from studies that were conducted in specialty settings, and aspects of the studied telehealth interventions were often incompletely described. However, much of the long-term management of these chronic conditions occurs within the context of primary care settings. As primary care settings likely have different pressures and challenges with telehealth modalities, given the need to address multiple comorbidities during the same visit, the results from our review may not be directly applicable. Thus, we recommend future reviews to examine and provide evidence-based guidance about the effect of telehealth interventions to deliver high-quality care using the right modality for the right patients with the right clinical condition at the right time.

### Additional Approaches to Examine Telehealth

A way to determine the effect telehealth is to use noninferiority analytic approaches when hypotheses focus on whether telehealth delivered care is equally effective to in-person care. Our eligibility criteria focused on randomized controlled trials and did not include observational study designs. Randomized controlled trials are the gold standard; however, conducting these trials is time-consuming and resource-intensive. Importantly, findings from randomized controlled trials take years to affect clinical practice, if they are implemented at all. Randomized controlled trials should not be expected to fill all the research gaps in the implementation and adoption of telehealth for chronic disease management. Thus, given the paucity of randomized controlled trials, we strongly recommend that future reviews focused on telehealth include what are likely to be rich and robust, but potentially biased; observational; and alternatively designed studies that emerge during and after the COVID-19 pandemic.

### Future Directions

Overall, there are 5 key areas in which future studies on this topic can fill the existing gaps and improve the approach. First, and perhaps most critical, telehealth interventions should be thoroughly described to maximize reproducibility and generalizability in other clinical contexts. Guidance exists on mobile and web-based interventions, which may provide indirect suggestions about key characteristics for telehealth intervention description. Second, there is a need to evaluate how best to integrate telehealth as a replacement for in-person care. Furthermore, there is a need to assess which clinical settings are best suited to the telehealth environment (eg, primary care vs specialty care settings). Approaches to integrating telehealth can be expected to vary across settings with different workflow patterns, clinical resources, and competing clinical demands, which emphasizes the need for solid evidence. Third, outcomes varied across the included studies, and some important outcomes were not addressed by any study (eg, impact on clinical workflow, patient satisfaction with telehealth experience, and subsequent use). Fourth, investigators should be encouraged to consider a priori subgroup evaluations or make individual patient-level data available, so that future reviews can identify patient-level characteristics associated with better outcomes with telehealth. Finally, future studies should also actively solicit and report patient perspectives and feedback on telehealth interventions to better inform intervention design. Such information can guide clinics and health care systems to offer optimal patient-centered telehealth delivery and support efforts to ensure equitable benefits and access to telehealth.

### Strengths and Limitations

Our review benefited from being protocol-driven, leveraging input from an expert panel consisting of clinicians and telehealth researchers, identifying disease-specific clinical outcomes, using an analytic framework to guide the understanding of telehealth modalities, and using a detailed approach to categorize and define telehealth components in chronic disease self-management. In addition, our review was based on a clear definition and use of telehealth. Notably, we acknowledge that individual patient characteristics (eg, race and ethnicity, gender, age, and rural status) may moderate the relationship between the modality in which the clinical visit occurs and any clinical-level and system-level outcomes.

Despite these strengths, our approach had some limitations. First, we included only the studies that met the EPOC criteria in this review; however, observational studies may have findings relevant to the provision of synchronous telehealth for chronic illness management. However, we do not believe that this limitation largely affected our findings. Second, we focused this review on 3 of the most prevalent chronic diseases, but there may be appropriately designed studies that targeted other conditions that we did not include. Third, we only included studies conducted in OECD countries, and thus, we may have missed relevant studies conducted in other countries. Fourth, given the small number of studies that we identified, statistical methods to detect publication bias were not conducted. Although it is possible that individual health systems or clinics have conducted quality improvement studies evaluating differences in experiences between synchronous and in-person care—especially during the COVID-19 pandemic—we suspect it to be unlikely that studies meeting EPOC criteria on this intervention have not been published, given the recent emphasis on the role of telehealth. Fifth, we identified few studies overall, and most studies had <100 patients and were assessed as having unclear or high ROB. Intervention core components, intervention fidelity, and impact of intervention on clinical workflow were not reported in any study. In addition, the interactions between clinicians and patients during telehealth episodes were not adequately or explicitly described, and most of our outcomes of interest were not consistently reported across the studies. These omissions limited the interpretation and replication of the evaluated interventions. Sixth, the included telehealth interventions used different telehealth modalities (email, phone, and video) with different hardware, delivered via different numbers of clinical interactions between patients and clinicians, over a wide range of intervention durations, and within different health care systems, which inherently make comparison between them challenging. Finally, the studies included in our review did not specify how they used or defined gender (man, woman, or nonbinary) or sex (male, female, or intersex) in their publications. Information on gender and sex is important to be captured and described for telehealth studies and research. Future studies should consider including observational studies; studies on additional, highly prevalent chronic diseases; studies conducted in non-OECD countries; and studies that do not meet the EPOC criteria especially, as those conducted since the onset of the COVID-19 pandemic may provide useful information.

### Conclusions

The COVID-19 pandemic precipitated a rapid shift from in-person to telehealth delivery, without a clear understanding about the impacts of telehealth on important health outcomes. Previous studies have found that telehealth modalities can improve health outcomes through the supplementation of in-person management of certain chronic diseases, particularly with approaches such as remote monitoring and patient education. However, we found that, currently, there is very little evidence on the use of telehealth as a replacement for in-person care for several chronic conditions and that the studies in this area remain insufficient and methodologically inconsistent. In conclusion, our review builds on this existing body of literature by evaluating the comparative literature on the effectiveness of telehealth visits delivered as a substitute for in-person visits for chronic disease management and provides recommendations for future studies in this area.

## Appendix

Multimedia Appendix 1 Subject headings and key words used in the search for relevant literature.

Multimedia Appendix 2 Study characteristics.

Multimedia Appendix 3 Intervention characteristics.

Multimedia Appendix 4 All outcomes reported in the included studies.

Multimedia Appendix 5 Excluded studies and the reason for exclusion.

Multimedia Appendix 6 Ad hoc horizon scan to identify relevant studies in progress.

## Abbreviations

ALT: alanine aminotransferase
CHF: congestive heart failure
COPD: chronic obstructive pulmonary disease
EPOC: Effective Practice and Organization of Care
HbA_1c_: glycosylated hemoglobin
OECD: Organisation for Economic Co-operation and Development
PRISMA: Preferred Reporting Items for Systematic Reviews and Meta-Analyses
PROSPERO: International Prospective Register of Systematic Reviews
ROB: risk of bias
T2DM: type 2 diabetes mellitus
VHA: Veterans Health Administration
