# Supplementary material for: Telehealth for the Longitudinal Management of Chronic Conditions: Systematic Review
Source: J Med Internet Res. 2022 Aug 26;24(8):e37100. doi: 10.2196/37100 (PMC9463619; doi:10.2196/37100)
Supplement: Multimedia Appendix 1 [file jmir_v24i8e37100_app1.docx]

**Multimedia Appendix 1.** Subject headings and keywords used in search of relevant literature.

**Database: MEDLINE (via Ovid MEDLINE(R) ALL 1946 to February 5, 2021)**

**Search Date: 2/7/2021**

| **Search Set** | **Search Strategy** | **Results** |
| --- | --- | --- |
| #1  *Virtual Care terms* | exp Telemedicine/ or exp Remote Consultation/ or Videoconferencing/ or  Telephone/ or exp Cell Phone/ or exp Computers, Handheld/ or (virtual or virtually or telehealth or tele-health or telemedicine or tele-medicine or telemedical or tele-medical or telecare or tele-care or teleconsult* or tele-consult* or telecommunicat* or tele-communicat* or telemanag* or tele-manag* or telehome or tele-home or telepharmac* or tele-pharmac* or telecardiol* or tele-cardiol* or tele-cardiac or teleintervention* or tele-intervention* or teleconferenc* or tele-conferenc* or telephon* or tele-phon* or cellphon* or cell-phon* or smartphon* or "mobile phone" or "mobile phones" or e-visit* or evisit* or e-care or ecare or e-consult* or econsult* or e-diagnos* or ediagnos* or e-medicine or emedicine or e-physician* or ephysician* or eclinician* or e-clinician* or e-pharm* or epharm* or "communication technology" or "communication technologies" or eHealth or e- health or "e health" or mHealth or m-health or "m health").ti,ab. | 271,845 |
| #2  *Virtual care terms, cont.* | ((mobile or digital) adj health*).ti,ab. | 6,153 |
| #3  *Virtual care terms, cont* | ((videoconferenc* or video-conferenc* or webconferenc* or web-conferenc* or webex or zoom or skype or ooVoo or FaceTime or Tango or GoToMeeting or "web based" or web-based or webbased) adj2 health*).ti,ab. | 711 |
| #4  *Virtual care terms, cont.* | (tele adj (care or diagnos* or health* or intervention* or manag* or therap* or treat* or medicine or medical or prescrib* or prescript*)).ti,ab. | 404 |
| #5  *Virtual care terms, cont.* | ((remote* or video* or internet or web or online) adj2 (meet* or call* or chat* or conferenc* or consult* or care or counsel* or visit*)).ti,ab. | 8,431 |
| #6  *combining* | 1 or 2 or 3 or 4 or 5 | 279,398 |
| #7  *HF terms* | exp Heart Failure/ or (CHF or CCF or HFpEF or HFrEF or "systolic dysfunction" OR "diastolic dysfunction").ti,ab. | 144,721 |
| #8  *HF terms, cont.* | ((heart or cardiac or cardiogenic) adj1 (failure or shock or arrest)).ti,ab. | 226,892 |
| #9  *HF terms, cont.* | ((preserved or reduced) adj2 "ejection fraction").ti,ab. | 9,110 |
| #10  *T2DM terms* | exp Diabetes Mellitus, Type 2/ or (DM or DM2 or DMii or T2D or T2DM or NIDDM or IDDM or MODY).ti,ab. | 199,623 |
| #11  *T2DM terms, cont.* | (diabet* adj2 ("type 2" or "type two" or II or "adult onset" or adult-onset or noninsulin or "non insulin" or non-insulin or maturity-onset or "maturity onset" or "slow onset" or slow-onset)).ti,ab. | 165,479 |
| #12  *COPD terms* | exp Pulmonary Disease, Chronic Obstructive/ or (COPD or COAD or emphysema*).ti,ab. | 89,548 |
| #13  *COPD terms, cont.* | (obstruct* adj2 (pulmonary or lung* or airflow* or airway* or bronch* or respirat*)).ti,ab. | 87,246 |
| #14  *COPD terms, cont.* | (chronic adj2 bronchit*).ti,ab. | 11,051 |
| #15  *combining* | 7 or 8 or 9 or 10 or 11 or 12 or 13 or 14 | 653,893 |
| #16  *combining* | 6 and 15 | 7,731 |
| #17  *Animal-only study exclusion* | 16 not (exp animals/ not exp humans/) | 7,532 |
| #18  *Population exclusion* | 17 not ((exp adolescent/ or exp child/ or exp infant/) not exp adult/) | 7,345 |
| #19  *Study designs* | exp Evaluation Studies as Topic/ or exp Cohort Studies/ or exp Longitudinal Studies/ or randomized controlled trial.pt. or controlled clinical trial.pt. or comparative study.pt. or clinical trial.pt. or evaluation study.pt. or (randomized or randomised or randomization or randomisation or placebo or randomly or trial or groups or "clinical trial" or "clinical trials" or "evaluation study" or "evaluation studies" or "intervention study" or "intervention studies" or cohort or longitudinal or longitudinally or prospective or prospectively or "follow up" or "comparative study" or "comparative studies" or nonrandom or "non-random" or nonrandomized or "non-randomized" or nonrandomised or "non-randomised" or quasi-experiment* or quasiexperiment* or quasirandom* or quasi-random* or quasi-control* or quasicontrol* or "pre-post" or posttest or "post-test" or pretest or "pre-test" or "repeated measure" or "repeated measures").ti,ab. | 7,831,571 |
| #20  *Study designs* | (before and after).ti,ab. | 771,878 |
| #21  *Study designs* | (before and during).ti,ab. | 403,950 |
| #22  *Study designs* | ("time series" and interrupt*).ti,ab. | 3,697 |
| #23  *Study designs* | ("time points" and (multiple or one or two or three or four or five or six or seven or eight or nine or ten or month or monthly or day or daily or week or weekly or hour or hourly)).ti,ab. | 69,056 |
| #24 | 19 or 20 or 21 or 22 or 23 | 8,265,282 |
| #25 | 18 and 24 | 4,784 |

**Database: EMBASE (via Elsevier)**

Search date: 2/7/2021
*Note: search from the Results page*

| **Search Set** | **Search Strategy** | **Results** |
| --- | --- | --- |
| #1  *Virtual Care terms* | 'telemedicine'/exp OR 'teleconsultation'/exp OR 'videoconferencing'/exp OR 'telephone'/exp OR 'mobile phone'/exp OR 'personal digital assistant'/exp OR (virtual OR virtually OR telehealth OR tele-health OR telemedicine OR tele-medicine OR telemedical OR tele-medical OR telecare OR tele-care OR teleconsult* OR tele-consult* OR telecommunicat* OR tele-communicat* OR telemanag* OR tele-manag* OR telehome OR tele-home OR telepharmac* OR tele-pharmac* OR telecardiol* OR tele-cardiol* OR tele-cardiac OR teleintervention* OR tele-intervention* OR teleconferenc* OR tele-conferenc* OR telephon* OR tele-phon* OR cellphon* OR cell-phon* OR smartphon* OR smart-phon* OR 'mobile phone' OR 'mobile phones' OR e-visit* OR evisit* OR e-care OR ecare OR e-consult* OR econsult* OR e-diagnos* OR ediagnos* OR e-medicine OR emedicine OR e-physician* OR ephysician* OR eclinician* OR e-clinician* OR e-pharm* OR epharm* OR 'communication technology' OR 'communication technologies' OR eHealth OR e- health OR 'e health' OR mHealth OR m-health OR 'm health'):ti,ab | 324,073 |
| #2  *Virtual care terms, cont.* | ((mobile OR digital) NEAR/1 health*):ti,ab | 6,902 |
| #3  *Virtual care terms, cont* | ((videoconferenc* OR video-conferenc* OR webconferenc* OR web-conferenc* OR webex OR zoom OR skype OR ooVoo OR FaceTime OR Tango OR GoToMeeting OR 'web based' OR web-based OR webbased) NEAR/2 health*):ti,ab | 767 |
| #4  *Virtual care terms, cont.* | (tele NEAR/1 (care OR diagnos* OR health* OR intervention* OR manag* OR therap* OR treat* OR medicine OR medical OR prescrib* OR prescript*)):ti,ab | 896 |
| #5  *Virtual care terms, cont.* | ((remote* OR video* OR internet OR web OR online) NEAR/2 (meet* OR call* OR chat* OR conferenc* OR consult* OR care OR counsel* OR visit*)):ti,ab | 11,958 |
| #6  *combining* | #1 OR #2 OR #3 OR #4 OR #5 | 334,175 |
| #7  *HF terms* | 'heart failure'/exp OR (CHF OR CCF OR HFpEF OR HFrEF OR 'systolic dysfunction' OR 'diastolic dysfunction'):ti,ab | 570,806 |
| #8  *HF terms, cont.* | ((heart OR cardiac OR cardiogenic) NEAR/1 (failure OR arrest OR shock)):ti,ab | 383,299 |
| #9  *HF terms, cont.* | ((preserved OR reduced) NEAR/2 'ejection fraction'):ti,ab | 18,518 |
| #10  *T2DM terms* | 'non insulin dependent diabetes mellitus'/exp OR (DM OR DM2 OR DMii OR T2D OR T2DM OR NIDDM OR IDDM OR MODY):ti,ab | 360,063 |
| #11  *T2DM terms, cont.* | (diabet* NEAR/2 ('type 2' OR 'type two' OR II OR 'adult onset' OR adult-onset OR noninsulin OR 'non insulin' OR non-insulin OR maturity-onset OR 'maturity onset' OR 'slow onset' OR slow-onset)):ti,ab | 249,817 |
| #12  *COPD terms* | 'chronic obstructive lung disease'/exp OR (COPD OR COAD OR emphysema*):ti,ab | 196,655 |
| #13  *COPD terms, cont.* | (obstruct* NEAR/2 (pulmonary OR lung* OR airflow* OR airway* OR bronch* OR respirat*)):ti,ab | 130,569 |
| #14  *COPD terms, cont.* | (chronic NEAR/2 bronchit*):ti,ab | 17,776 |
| #15  *combining* | #7 OR #8 OR #9 OR #10 OR #11 OR #12 OR #13 OR #14 | 1,242,111 |
| #16  *combining* | #6 AND #15 | 17,245 |
| #17  *Animal-only study exclusion* | #16 AND [humans]/lim | 15,940 |
| #18  *Population exclusion* | #17 NOT (([child]/lim OR [infant]/lim OR [newborn]/lim OR [preschool]/lim) NOT ([adult]/lim OR [middle aged]/lim OR [young adult]/lim)) | 15,600 |
| #19  *Study designs* | 'randomized controlled trial'/exp OR 'crossover procedure'/exp OR 'double blind procedure'/exp OR 'single blind procedure'/exp OR randomization:ti,ab OR randomisation:ti,ab OR randomized:ti,ab OR randomised:ti,ab OR randomly:ti,ab OR crossover:ti,ab OR 'cross over':ti,ab OR placebo:ti,ab OR 'double blind':ti,ab OR 'double blinded':ti,ab OR 'single blind':ti,ab OR 'single blinded':ti,ab OR 'clinical study'/exp OR 'clinical trial':ti,ab OR 'clinical trials':ti,ab OR 'controlled study'/exp OR 'evaluation study'/exp OR 'evaluation study':ti,ab OR 'evaluation studies':ti,ab OR 'intervention study'/exp OR 'intervention study':ti,ab OR 'intervention studies':ti,ab OR 'case control study'/exp OR 'case control':ti,ab OR 'cohort analysis'/exp OR cohort:ti,ab OR cohorts:ti,ab OR longitudinal:ti,ab OR longitudinally:ti,ab OR prospective:ti,ab OR prospectively:ti,ab OR retrospective:ti,ab OR 'follow up'/exp OR 'follow up':ti,ab OR 'comparative effectiveness'/exp OR 'comparative study'/exp OR 'comparative study':ti,ab OR 'comparative studies':ti,ab | 17,359,756 |
| #20  *Study designs* | 'pre post':ti,ab OR prepost:ti,ab OR 'post test':ti,ab OR posttest:ti,ab OR pretest:ti,ab OR 'pre test':ti,ab OR 'quasi experiment':ti,ab OR quasiexperiment:ti,ab OR 'quasi experimental':ti,ab OR quasiexperimental:ti,ab OR quasirandom:ti,ab OR 'quasi random':ti,ab OR 'quasi control':ti,ab OR quasicontrol:ti,ab OR 'repeated measure':ti,ab OR 'repeated measures':ti,ab | 132,600 |
| #21  *Study designs* | ('time series':ti,ab AND interrupt*:ti,ab) OR (before:ti,ab AND after:ti,ab) OR (before:ti,ab AND during:ti,ab) | 1,360,078 |
| #22  *Study designs* | 'time points':ti,ab AND (multiple:ti,ab OR one:ti,ab OR two:ti,ab OR three:ti,ab OR four:ti,ab OR five:ti,ab OR six:ti,ab OR seven:ti,ab OR eight:ti,ab OR nine:ti,ab OR ten:ti,ab OR month:ti,ab OR monthly:ti,ab OR day:ti,ab OR days:ti,ab OR daily:ti,ab OR week:ti,ab OR weekly:ti,ab OR hour:ti,ab OR hourly:ti,ab) | 115,754 |
| #23  *combining* | #19 OR #20 OR #21 OR #22 | 17,784,738 |
| #24  *combining* | #18 AND #23 | 11,324 |
| #25  *exclusions* | #24 NOT ('case report'/exp OR 'case study'/exp OR 'editorial'/exp OR [editorial]/lim OR 'letter'/exp OR [letter]/lim OR 'note'/exp OR [note]/lim OR [conference abstract]/lim OR 'conference abstract'/exp OR 'conference abstract'/it) | 6,461 |

**Database: Cochrane Central Register of Controlled Trials (via Ovid)**
Search date: 6/29/2021
*Note: through May 2021*

| **Search Set** | **Search Strategy** | **Results** |
| --- | --- | --- |
| #1  *Virtual Care terms* | exp Telemedicine/ or exp Remote Consultation/ or Videoconferencing/ or  Telephone/ or exp Cell Phone/ or exp Computers, Handheld/ or (virtual or virtually or telehealth or tele-health or telemedicine or tele-medicine or telemedical or tele-medical or telecare or tele-care or teleconsult* or tele-consult* or telecommunicat* or tele-communicat* or telemanag* or tele-manag* or telehome or tele-home or telepharmac* or tele-pharmac* or telecardiol* or tele-cardiol* or tele-cardiac or teleintervention* or tele-intervention* or teleconferenc* or tele-conferenc* or telephon* or tele-phon* or cellphon* or cell-phon* or smartphon* or "mobile phone" or "mobile phones" or e-visit* or evisit* or e-care or ecare or e-consult* or econsult* or e-diagnos* or ediagnos* or e-medicine or emedicine or e-physician* or ephysician* or eclinician* or e-clinician* or e-pharm* or epharm* or "communication technology" or "communication technologies" or eHealth or e- health or "e health" or mHealth or m-health or "m health").ti,ab. | 44,030 |
| #2  *Virtual care terms, cont.* | ((mobile or digital) adj health*).ti,ab. | 1,594 |
| #3  *Virtual care terms, cont* | ((videoconferenc* or video-conferenc* or webconferenc* or web-conferenc* or webex or zoom or skype or ooVoo or FaceTime or Tango or GoToMeeting or "web based" or web-based or webbased) adj2 health*).ti,ab. | 277 |
| #4  *Virtual care terms, cont.* | (tele adj (care or diagnos* or health* or intervention* or manag* or therap* or treat* or medicine or medical or prescrib* or prescript*)).ti,ab. | 182 |
| #5  *Virtual care terms, cont.* | ((remote* or video* or internet or web or online) adj2 (meet* or call* or chat* or conferenc* or consult* or care or counsel* or visit*)).ti,ab. | 3,208 |
| #6  *combining* | 1 or 2 or 3 or 4 or 5 | 46,651 |
| #7  *HF terms* | exp Heart Failure/ or (CHF or CCF or HFpEF or HFrEF or "systolic dysfunction" OR "diastolic dysfunction").ti,ab. | 14,236 |
| #8  *HF terms, cont.* | ((heart or cardiac or cardiogenic) adj1 (failure or shock or arrest)).ti,ab. | 33,892 |
| #9  *HF terms, cont.* | ((preserved or reduced) adj2 "ejection fraction").ti,ab. | 2,416 |
| #10  *T2DM terms* | exp Diabetes Mellitus, Type 2/ or (DM or DM2 or DMii or T2D or T2DM or NIDDM or IDDM or MODY).ti,ab. | 30,890 |
| #11  *T2DM terms, cont.* | (diabet* adj2 ("type 2" or "type two" or II or "adult onset" or adult-onset or noninsulin or "non insulin" or non-insulin or maturity-onset or "maturity onset" or "slow onset" or slow-onset)).ti,ab. | 42,140 |
| #12  *COPD terms* | exp Pulmonary Disease, Chronic Obstructive/ or (COPD or COAD or emphysema*).ti,ab. | 19,378 |
| #13  *COPD terms, cont.* | (obstruct* adj2 (pulmonary or lung* or airflow* or airway* or bronch* or respirat*)).ti,ab. | 16,735 |
| #14  *COPD terms, cont.* | (chronic adj2 bronchit*).ti,ab. | 1,877 |
| #15  *combining* | 7 or 8 or 9 or 10 or 11 or 12 or 13 or 14 | 109878 |
| #16  *combining* | 6 and 15 | 4,022 |
| #17  *Animal-only study exclusion* | 16 not (exp animals/ not exp humans/) | 4,022 |
| #18  *Population exclusion* | 17 not ((exp adolescent/ or exp child/ or exp infant/) not exp adult/) | 4,008 |
| #19  *Study designs* | exp Evaluation Studies as Topic/ or exp Cohort Studies/ or exp Longitudinal Studies/ or (randomized or randomised or randomization or randomisation or placebo or randomly or trial or groups or "clinical trial" or "clinical trials" or "evaluation study" or "evaluation studies" or "intervention study" or "intervention studies" or cohort or longitudinal or longitudinally or prospective or prospectively or "follow up" or "comparative study" or "comparative studies" or nonrandom or "non-random" or nonrandomized or "non-randomized" or nonrandomised or "non-randomised" or quasi-experiment* or quasiexperiment* or quasirandom* or quasi-random* or quasi-control* or quasicontrol* or "pre-post" or posttest or "post-test" or pretest or "pre-test" or "repeated measure" or "repeated measures").ti,ab. | 1,390,806 |
| #20  *Study designs* | ("time series" and interrupt*).ti,ab. | 395 |
| #21  *Study designs* | ("time points" and (multiple or one or two or three or four or five or six or seven or eight or nine or ten or month or monthly or day or daily or week or weekly or hour or hourly)).ti,ab. | 20,644 |
| #22 | 19 or 20 or 21 | 1,391,931 |
| #23 | 18 and 22 | 3,637 |
