# Supplementary material for: Telehealth for the Longitudinal Management of Chronic Conditions: Systematic Review
Source: J Med Internet Res. 2022 Aug 26;24(8):e37100. doi: 10.2196/37100 (PMC9463619; doi:10.2196/37100)
Supplement: Multimedia Appendix 2 [file jmir_v24i8e37100_app2.docx]

**Multimedia Appendix 2**. Detail on study characteristics

| **Study**  **Country**  **# Enrolled**  **# Arms**  **Funding Source**  **Companion Paper** | **Type of intervention**  **Frequency**  **Duration** | **Eligibility** | **Population**  **Mean Age (SD)**  **Female %**  **Race %**  **VA based** | **Outcomes Types** | **Risk of Bias for**  **Objective and Patient-Reported Outcomes** |
| --- | --- | --- | --- | --- | --- |
| *Congestive heart failure* | | | | | |
| Hansen,  2018^29^  Germany  210 patients  3 arms  Abbott | Remote monitoring +Telephone; Remote monitoring + in-person; Remote monitoring + automated telemetry follow-up  Quarterly  12 months | Inclusion criteria: (1) 18-80 years; (2) CHF^a^ w/ LVEF^b^ ≤ 35%, NYHA^c^ class I-III; (3) home infrastructure to support use of a home transmitter and s/p ICD^d^/CRT-D^e^ implantation (new, upgrade or generator replacement).  Exclusion criteria: (1) 2nd degree Mobitz type II AV block; (2) 3rd degree AV^f^ block; (3) severe renal insufficiency; (4) less than 1-year life expectancy; (5) pregnant; (6) already enrolled in a study; (7) MI^g^/ cardiac catheter within 3 months prior to the study. | Mean age: 65.1 (10.1)  Female: 14.8%  Race: NR^h^  Not VA based | NYHA class/symptoms  Hospitalization | Objective: Unclear  Patient reported: High |
| *Type 2 diabetes mellitus* | | | | | |
| Jeong,  2018^28^  South Korea  338 patients  3 arms  Korea Ministry of Health & Welfare | Remote Monitoring + video; In-person  3 times  24 weeks | Inclusion criteria: T2DM^h^ with A1c^i^ range 7-11%.  Exclusion criteria: (1) using insulin (basal or premixed insulin) more than twice a day; (2) unable to use a personal computer to access the Internet at home; (3) acute illness, liver dysfunction, renal dysfunction, or chronic lung disease, or any other medical conditions that could affect glycemic level. | Mean age: 53 (9.10)  Female: 33%  Race: NR  Not VA based | A1c  Emergency room visits  Hospitalization | Objective: Low  Patient reported: Low |
| Klingeman,  2017^25^  USA  60 patients  2 arms  University of Michigan | Telephone and e-mail monitoring; In-person  Variable number of contacts per patient  1 year | Inclusion criteria: (1) adults w/ T2DM w/ 3+ diabetes meds +/- insulin; (2) A1c >/= 8%, </= 11; (3) able and willing to use telephonic communication regularly between visits; and (4) new patients prior to first visit to the endocrinology clinic.  Exclusion criteria: (1) non-English speakers; (2) patients already treated by an endocrinologist; (3) shortened life expectancy. | Mean age: 54.4 (9.6)  Female: 47%  Race: 87% White; 10% Black; 2% Hispanic; 2% Other  Not VA based | A1c  ER visits  Hospitalization | Objective: High  Patient reported: High |
| Rasmussen, 2016^30^  Denmark  40 patients  2 arms  Danish National Health Department | Video visits conducted via specialized equipment (TandBerg E20)  3 weeks | Inclusion criteria: (1) live at home; (2) able to communicate by video telephone; (3) no psychiatric disorders; (4) age 40–85 years; (5) able to administer medication themselves.  Exclusion criteria: (1) type 1 diabetes mellitus; (2) speech disabilities; (3) non-Danish speakers; (4) severe chronic disease (renal failure, liver insufficiency, current cancer treatment). | Median age: 62.7  Female: 32%  Race: 100% White  Not VA based | A1c | Objective: Low  Patient reported: NA |
| Whitlock,  2000^1^  USA  28 patients  2 arms  Department of Defense | Video  telemedicine was delivered via the Aviva tele care equipment which included a blood pressure meter, an electronic stethoscope.  Nurse case manager contact once a week and physician contact once a month  3 months | Inclusion criteria: (1) adults with a A1c >8%; (2) diagnosis of T2DM.  Exclusion criteria: (1) inability to use equipment; (2) pending surgery; (3) documented psychiatric history; (4) A1c <8.0%. | Mean age: 63 (NR)  Female: 61%  Race: NR  Not VA based | A1c | Objective: High  Patient reported: Unclear |

^a^CHF = congestive heart failure

^b^LVEF = left ventricular ejection fraction

^c^NYHA= New York Heart Association

^d^ICD = implanted cardioverter defibrillator

^e^CRT -D = cardiac resynchronization therapy-defibrillator

^f^AV = atrioventricular

^g^MI = myocardial infarction

^h^T2DM = type 2 diabetes mellitus

^i^A1c = Hemoglobin A1c

^j^NR = not reported
