# Supplementary material for: Telehealth for the Longitudinal Management of Chronic Conditions: Systematic Review
Source: J Med Internet Res. 2022 Aug 26;24(8):e37100. doi: 10.2196/37100 (PMC9463619; doi:10.2196/37100)
Supplement: Multimedia Appendix 3 [file jmir_v24i8e37100_app3.docx]

**Multimedia Appendix 3.** Detail on intervention characteristics

| **Author, Year**  **# Enrolled**  **# Arms** | **Intervention description** | **Mode of intervention**  **Platform**  **Type of Clinician(s)** | **Frequency of contacts**  **Duration of contact** | **Data available at the time of the virtual interaction** | **Comparator** |
| --- | --- | --- | --- | --- | --- |
| *Congestive heart failure* | | | | | |
| Hansen  2018 ^29^  210 patients  3 arms | Patients with CHF^a^ followed for 12 months between first and 13th month post-implantation of ICD^b^/CRT-D^c^ - one arm with remote telemetry monitoring with automated quarterly follow-up only, and then a second arm in which patients received personal, scheduled follow-up quarterly. Personal contact arm randomized to phone vs in-person contact for follow-up (comparison of interest). | Remote monitoring + telephone; Remote monitoring + in-person; Remote monitoring + automated follow-up  Remote monitoring and automated follow-up were reported via Merlin.net and “Merlin@Home”^TM^ transmitter  Cardiologist | 4 phone contacts; 4 face-to-face contacts  12 months | ICD/CRT-D telemetry data | Arm 1: Remote monitoring + in-person, previously scheduled visits; Arm 2: Remote monitoring +automated follow-up |
| *Type 2 diabetes mellitus* | | | | | |
| Jeong,  2018 ^28^  338 patients  3 arms | Three arms (1 usual care, 2 active intervention); comparison of interest among two intervention arms  Telemonitoring group: involves asynchronous transmission of home glucose values via "Smart Care Unit" and receives automated responses by algorithm and weekly general diabetes mellitus education with in-person follow-up on 8,16,24 weeks  Telemedicine group: involves telemonitoring as described but follow-up with endocrinology were by video at weeks 8 and 16 while 24-week follow-up was in person. | Video; In-person  Smart care unit: personal tablet with abilities to: (1) video conference and text message endocrinologist; (2) auto-transmit blood glucose data from patient’s glucometer; (3) provide additional information to support diabetes self-care.  Endocrinologist | 8,16,24 weeks  24 weeks | Remote monitoring home glucose values; body composition analyzer | Arm 1: Conventional care; Arm 2: telemonitoring with all visits in-person |
| Klingeman,  2017 ^25^  60 patients  2 arms | The intervention consisted of endocrinology clinic-initiated and pre-scheduled phone calls or e-mails; frequency of interaction was tailored to each patient. Interactions consisted of reviewing glucose readings and monitoring blood pressure. Ad hoc clinic visits could be added as indicated, and pre-scheduled contact intervals adjusted. | Telephone; In-person; e-mail  Endocrinologist | Variable "tailored" per patient  1 year | BP^d^, glucose checks | Usual In-person care |
| Rasmussen, 2016^30^  40 patients  2 arms | Tested home treatment of T2DM^e^ by video consultation versus standard outpatient care. Patients who completed higher-level T2DM care with an Endocrinologist for poor metabolic control were transferred back to their GP^f^ at the completion of this care (usually 3 weeks). The intervention consisted of video consultations alternating between the clinician or nurse and patient. The control group attended outpatient visits. | Video  Videophone (model TandBerg E20)  Endocrinologist; Nurses | NR  3 weeks | BP | Usual In-person care |
| Whitlock,  2000^1^  28 patients  2 arms | Over a 3-month study period, the intervention group received weekly telemonitoring (voice and video interaction) visits by the case manager and once a month physician telemedicine (voice and video interaction) visits compared to the control group which received usual care. For intervention participants, the case manager, internist, and family practitioner emailed about the patient’s status, progress, and medication. | Video  Video system: Aviva 20/20 and then 10/10  Internist, family practitioner, case manager | Nurse case manager contact once a week and physician contact once a month  3 months | Data from case manager: blood glucose readings, blood pressure, weight, hypoglycemic episodes | Usual In-person care |

^a^CHF = congestive heart failure

^d^ICD = implanted cardioverter defibrillator

^e^CRT -D = cardiac resynchronization therapy-defibrillator

^d^BP = blood pressure

^e^T2DM = type 2 diabetes mellitus

^f^GP = general practitioner
