# Supplementary material for: Telehealth for the Longitudinal Management of Chronic Conditions: Systematic Review
Source: J Med Internet Res. 2022 Aug 26;24(8):e37100. doi: 10.2196/37100 (PMC9463619; doi:10.2196/37100)
Supplement: Multimedia Appendix 4 [file jmir_v24i8e37100_app4.docx]

**Multimedia Appendix 4.** all outcomes reported in included studies

| Study | Outcomes reported |
| --- | --- |
| *Type 2 diabetes mellitus* | |
| Jeong,  2018^28^ | - Change in A1c |
| Klingeman,  2017^25^ | - Change in A1c |
| Rasmussen, 2016^30^ | - Change in A1c |
| Whitlock,  2000^1^ | - Change in A1c |
| Klingeman,  2017^25^ | - Hospitalization |
| Klingeman,  2017^25^ | - Hospitalization |
| Jeong,  2018^28^ | - ED attendance |
| Klingeman,  2017^25^ | - ED attendance |
| *Congestive heart failure* | |
| Hansen,  2018^29^ | - NYHA class/symptoms |
| Hansen,  2018^29^ | - Hospitalization |
| *Harms* | |
| Jeong,  2018^28^ | - Adverse events - Death |
| Klingeman,  2017^25^ | - Hypoglycemia |
| Other utilization outcomes | |
| Hansen,  2018^29^ | - Unscheduled follow-ups - Proportion of all follow-ups that had disease-relevant findings |
| Klingeman,  2017^25^ | - Additional diabetes education - Face-to-face visits - Phone calls - Emails |
| Rasmussen, 2016^30^ | - Consultations |
| *Other clinical outcomes* | |
| Hansen,  2018 ^29^ | - Arrhythmias - Number of delivered/appropriate ICD Therapies - Changes in QoL - All-cause mortality |
| Jeong,  2018 ^28^ | - Frequency of hypoglycemia - Changes in fasting blood glucose - Lipid profiles - Body weight - BMI - Percent achieving goal HbA1c - Compliance with medications - Compliance with self-monitoring of blood glucose - Labs: aspartate aminotransferase (AST); alanine aminotransferase (ALT) Creatinine |
| Klingeman,  2017^25^ | - Statin use - Insulin use - Foot ulcers - Blood pressure - BMI |
| Rasmussen, 2016^30^ | - Mean glucose - Systolic blood pressure - Diastolic blood pressure - Cholesterol - Low-density-lipoprotein cholesterol (LDL) - Weight |
| Whitlock,  2000^1^ | - Total body weight - Microalbumin - Creatinine - Triglycerides - Low density lipoproteins |
| *Other outcomes* | |
| Whitlock,  2000^1^ | - Diabetes Quality of Life (DQOL) survey and Medical Outcome Study Health Survey (SF36) - Clinician Survey (limited results reported in this paper) |
