# Supplementary material for: Telehealth for the Longitudinal Management of Chronic Conditions: Systematic Review
Source: J Med Internet Res. 2022 Aug 26;24(8):e37100. doi: 10.2196/37100 (PMC9463619; doi:10.2196/37100)
Supplement: Multimedia Appendix 5 [file jmir_v24i8e37100_app5.docx]

**Multimedia Appendix 5.** details on excluded studies and the reason for exclusion

|  | Exclusion Reason | | | | | |
| --- | --- | --- | --- | --- | --- | --- |
| Study | Not OECD | Not Population | Not Intervention | Not Outcomes | Not Comparator | Not Design |
| Antonicelli, 2010^1^ |  |  | X |  |  |  |
| Basudev, 2016^2^ |  |  | X |  |  |  |
| Bekelman, 2015^3^ |  |  | X |  |  |  |
| Benatar, 2003^4^ |  |  | X |  |  |  |
| Bentley, 2014^5^ |  |  | X |  |  |  |
| Berkhof, 2015^6^ |  |  | X |  |  |  |
| Biermann, 2000^7^ |  | X |  |  |  |  |
| Blumenthal, 2014^8^ |  |  | X |  |  |  |
| Bowles, 2009^9^ |  |  | X |  |  |  |
| Brandon, 2009^10^ |  |  | X |  |  |  |
| Carral, 2015^11^ |  | X |  |  |  |  |
| Cartwright, 2013^12^ |  |  | X |  |  |  |
| Chen, 2019^13^ | X |  |  |  |  |  |
| Chen, 2011^14^ | X |  |  |  |  |  |
| Choe, 2005^15^ |  |  | X |  |  |  |
| Chwalow, 1989^16^ |  |  | X |  |  |  |
| Clifford, 2005^17^ |  |  | X |  |  |  |
| Cohen, 2020^18^ |  |  |  |  | X |  |
| Comin-Colet, 2016^19^ |  |  | X |  |  |  |
| Creason, 2001^20^ |  |  | X |  |  |  |
| Cui, 2013^21^ |  |  | X |  |  |  |
| Dadosky, 2018^22^ |  |  | X |  |  |  |
| Dale, 2007^23^ |  |  |  |  |  | X |
| Dansky, 2008^24^ |  |  | X |  |  |  |
| Dansky, 2009^25^ |  |  | X |  |  |  |
| de la Porte, 2007^26^ |  |  | X |  |  |  |
| De Simone, 2015^27^ |  |  | X |  |  |  |
| de Vries, 2011^28^ |  |  | X |  |  |  |
| Dienstl, 2011^29^ |  |  |  |  |  | X |
| Dixon, 2020^30^ |  |  |  |  | X |  |
| Doyle, 2017^31^ |  |  | X |  |  |  |
| Durso, 2003^32^ |  |  | X |  |  |  |
| Egede, 2018^33^ |  |  | X |  |  |  |
| Egede, 2017^34^ |  |  | X |  |  |  |
| Ell, 2012^35^ |  | X |  |  |  |  |
| Farrero, 2001^36^ |  |  | X |  |  |  |
| Farsaei, 2011^37^ | X |  |  |  |  |  |
| Gamez-Lopez, 2012^38^ |  |  | X |  |  |  |
| Gellis, 2012^39^ |  |  | X |  |  |  |
| González-Guerrero, 2018^40^ |  |  | X |  |  |  |
| Gorodeski, 2020^41^ |  |  | X |  |  |  |
| Hallberg, 2018^42^ |  |  | X |  |  |  |
| Hansen, 2017^43^ |  |  | X |  |  |  |
| Haynes, 2020^44^ |  |  | X |  |  |  |
| Herold, 2018^45^ |  |  | X |  |  |  |
| Holmen, 2016^46^ |  |  | X |  |  |  |
| Hsu, 2016^47^ |  |  | X |  |  |  |
| Huizinga, 2010^48^ |  |  | X |  |  |  |
| Inoriza, 2017^49^ |  |  | X |  |  |  |
| Jakobsen, 2015^50^ |  | X |  |  |  |  |
| Jakobsson, 2015^51^ |  | X |  |  |  |  |
| Jerant, 2003^52^ |  |  | X |  |  |  |
| Jimenez-Marrero, 2020^53^ |  |  | X |  |  |  |
| Kashem, 2008^54^ |  |  | X |  |  |  |
| Kashem, 2006^55^ |  |  | X |  |  |  |
| Kaur, 2015^56^ | X |  |  |  |  |  |
| Kessler, 2018^57^ |  |  | X |  |  |  |
| King, 2009^58^ |  |  | X |  |  |  |
| Kobb, 2003^59^ |  | X |  |  |  |  |
| Koehler, 2018^60^ |  |  | X |  |  |  |
| Koehler, 2011^61^ |  |  | X |  |  |  |
| Koehler, 2012^62^ |  |  | X |  |  |  |
| Krein, 2004^63^ |  |  | X |  |  |  |
| LaFramboise, 2003^64^ |  |  | X |  |  |  |
| Lam, 2011^65^ |  |  |  |  | X |  |
| Lauffenburger, 2019^66^ |  |  |  |  | X |  |
| Lauffenburger, 2019^67^ |  |  |  |  | X |  |
| Layman, 2020^68^ |  |  | X |  |  |  |
| Lehmann, 2006^69^ |  |  | X |  |  |  |
| Leichter, 2013^70^ |  | X |  |  |  |  |
| Lilholt, 2017^71^ |  |  | X |  |  |  |
| Liou, 2014^72^ | X |  |  |  |  |  |
| Litke, 2018^73^ |  |  |  |  | X |  |
| Lopez Cabezas, 2006^74^ |  |  | X |  |  |  |
| Lyons, 2016^75^ |  | X |  |  |  |  |
| Majithia, 2020^76^ |  |  |  |  | X |  |
| Martinez, 2013^77^ |  |  |  | X |  |  |
| Mayes, 2010^78^ |  |  | X |  |  |  |
| McElroy, 2016^79^ |  | X |  |  |  |  |
| Moayeri, 2019^80^ |  |  | X |  |  |  |
| Moore, 2017^81^ |  | X |  |  |  |  |
| Morguet, 2008^82^ |  | X |  |  |  |  |
| Mortara, 2009^83^ |  |  | X |  |  |  |
| Moyer-Knox, 2004^84^ |  |  |  |  |  | X |
| Myers, 2020^85^ |  |  |  |  | X |  |
| Nakayama, 2020^86^ |  |  | X |  |  |  |
| Nguyen, 2008^87^ |  |  | X |  |  |  |
| Nield, 2012^88^ |  |  | X |  |  |  |
| Nouryan, 2019^89^ |  |  | X |  |  |  |
| Odegard, 2005^90^ |  |  | X |  |  |  |
| Odeh, 2015^91^ |  |  | X |  |  |  |
| Oh, 2003^92^ |  |  | X |  |  |  |
| Pare, 2006^93^ |  |  | X |  |  |  |
| Pedone, 2015^94^ |  |  | X |  |  |  |
| Perez-Rodriguez, 2015^95^ |  |  | X |  |  |  |
| Polonsky, 2020^96^ |  |  |  |  | X |  |
| Quinn, 2016^97^ |  |  | X |  |  |  |
| Ringbaek, 2015^98^ |  |  | X |  |  |  |
| Rodriguez-Idigoras, 2009^99^ |  |  | X |  |  |  |
| Rüter, 2014^100^ |  |  | X |  |  |  |
| Salvo, 2012^101^ |  |  |  |  |  | X |
| Sarayani, 2018^102^ | X |  |  |  |  |  |
| Scalvini, 2005^103^ |  |  |  |  |  | X |
| Scalvini, 2006^104^ |  |  |  |  |  | X |
| Schmidt, 2019^105^ |  |  |  |  | X |  |
| Smith, 2008^106^ |  |  |  | X |  |  |
| Sorocco, 2013^107^ |  | X |  |  |  |  |
| Steventon, 2014^108^ |  |  | X |  |  |  |
| Stewart, 2015^109^ |  |  | X |  |  |  |
| Stone, 2010^110^ |  |  |  |  | X |  |
| Tabak, 2014^111^ |  |  | X |  |  |  |
| Taylor, 2009^112^ |  |  | X |  |  |  |
| Veenstra, 2015^113^ |  |  |  |  | X |  |
| Vidula, 2020^114^ |  |  | X |  |  |  |
| Vitacca, 2009^115^ |  | X |  |  |  |  |
| Wakefield, 2012^116^ |  |  | X |  |  |  |
| Wakefield, 2008^117^ |  |  | X |  |  |  |
| Whitten, 2007^118^ |  |  | X |  |  |  |
| Wild, 2016^119^ |  |  | X |  |  |  |
| Woodend, 2008^120^ |  |  | X |  |  |  |
| Wright, 2019^121^ |  |  | X |  |  |  |
| Wu, 2005^122^ |  |  | X |  |  |  |
| Yan, 2018^123^ | X |  |  |  |  |  |
| Yoo, 2009^124^ |  |  | X |  |  |  |

References to Multimedia Appendix 5

1. Antonicelli R, Mazzanti I, Abbatecola AM, Parati G. Impact of home patient telemonitoring on use of β-blockers in congestive heart failure. Drugs Aging 2010;27(10):801–805. PMID:20883060

**2.** Basudev N, Crosby-Nwaobi R, Thomas S, Chamley M, Murrells T, Forbes A. A prospective randomized controlled study of a virtual clinic integrating primary and specialist care for patients with Type 2 diabetes mellitus. Diabet Med 2016;33(6):768–776. PMID:27194175

**3.** Bekelman DB, Plomondon ME, Carey EP, Sullivan MD, Nelson KM, Hattler B, McBryde CF, Lehmann KG, Gianola K, Heidenreich PA, Rumsfeld JS. Primary results of the Patient-Centered Disease Management (PCDM) for heart failure study a randomized clinical trial. JAMA Intern Med 2015;175(5):725–732. PMID:25822284

**4.** Benatar D, Bondmass M, Ghitelman J, Avitall B. Outcomes of chronic heart failure. Arch Intern Med 2003;163(3):347–352. PMID:12578516

**5.** Bentley CL, Mountain GA, Thompson J, Fitzsimmons DA, Lowrie K, Parker SG, Hawley MS. A pilot randomised controlled trial of a Telehealth intervention in patients with chronic obstructive pulmonary disease: challenges of clinician-led data collection. Trials 2014;15(1):313. PMID:25100550

**6.** Berkhof FF, Van Den Berg JWK, Uil SM, Kerstjens HAM. Telemedicine, the effect of nurse-initiated telephone follow up, on health status and health-care utilization in COPD patients: a randomized trial. Respirology 2015;20(2):279–285. PMID:25400242

**7.** Biermann E, Dietrich W, Standl E. Telecare of diabetic patients with intensified insulin therapy. Stud Health Technol Inform 2000. p. 327–332. PMID:2001134190

**8.** Blumenthal JA, Emery CF, Smith PJ, Keefe FJ, Welty-Wolf K, Mabe S, Martinu T, Johnson JJ, Babyak MA, O’Hayer VF, Diaz PT, Durheim M, Baucom D, Palmer SM. The effects of a telehealth coping skills intervention on outcomes in chronic obstructive pulmonary disease: primary results from the INSPIRE-II study. Psychosom Med 2014;76(8):581–592. PMID:25251888

**9.** Bowles KH, Holland DE, Horowitz DA. A comparison of in-person home care, home care with telephone contact and home care with telemonitoring for disease management. J Telemed Telecare 2009;15(7):344–350. PMID:19815903

**10.** Brandon AF, Schuessler JB, Ellison KJ, Lazenby RB. The effects of an advanced practice nurse led telephone intervention on outcomes of patients with heart failure. Appl Nurs Res Appl Nurs Res; 2009;22(4):e1. PMID:19875032

**11.** Carral F, Ayala MDC, Fernández JJ, González C, Piñero A, García G, Cañavate C, Jiménez AI, García C. Web-based telemedicine system is useful for monitoring glucose control in pregnant women with diabetes. Diabetes Technol Ther 2015;17(5):349-354. PMID:25629547

**12.** Cartwright M, Hirani SP, Rixon L, Beynon M, Doll H, Bower P, Bardsley M, Steventon A, Knapp M, Henderson C, Rogers A, Sanders C, Fitzpatrick R, Barlow J, Newman SP. Effect of telehealth on quality of life and psychological outcomes over 12 months (Whole Systems Demonstrator telehealth questionnaire study): Nested study of patient reported outcomes in a pragmatic, cluster randomised controlled trial. BMJ 2013;346(7897). PMID:23444424

**13.** Chen C, Li X, Sun L, Cao S, Kang Y, Hong L, Liang Y, You G, Zhang Q. Post-discharge short message service improves short-term clinical outcome and self-care behaviour in chronic heart failure. ESC Hear Fail 2019;6(1):164–173. PMID:30478888

**14.** Chen SY, Chang YH, Hsu HC, Lee YJ, Hung YJ, Hsieh CH. One-year efficacy and safety of the telehealth system in poorly controlled type 2 diabetic patients receiving insulin therapy. Telemed J E Health 2011;17(9):683–687. PMID:21882998

**15.** Choe HM, Mitrovich S, Dubay D, Hayward RA, Krein SL, Vijan S. Proactive case management of high-risk patients with type 2 diabetes mellitus by a clinical pharmacist: A randomized controlled trial. Am J Manag Care 2005;11(4):253–260. PMID:15839185

**16.** Chwalow AJ, Costagliola D, Stern J, Mesbah M, Eschwege E. Telephone versus face to face interviewing as a means of collecting data relevant to the management of diabetes among general practitioners in France: a randomized design. Diabete Metab 1989;15(4):157–160. PMID:2806701

**17.** Clifford RM, Davis WA, Batty KT, Davis TME. Effect of a pharmaceutical care program on vascular risk factors in type 2 diabetes: the Fremantle Diabetes Study. Diabetes Care 2005;28(4):771–776. PMID:15793171

**18.** Cohen LB, Taveira TH, Wu WC, Pirraglia PA. Pharmacist-led telehealth disease management program for patients with diabetes and depression. J Telemed Telecare 2020;26(5):294–302. PMID:30691328

**19.** Comín-Colet J, Enjuanes C, Verdú-Rotellar JM, Linas A, Ruiz-Rodriguez P, González-Robledo G, Farré N, Moliner-Borja P, Ruiz-Bustillo S, Bruguera J. Impact on clinical events and healthcare costs of adding telemedicine to multidisciplinary disease management programmes for heart failure: Results of a randomized controlled trial. J Telemed Telecare 2016;22(5):282–295. PMID:26350543

**20.** Creason H. Congestive heart failure telemanagement clinic. Lippincotts Case Manag 2001;6(4):146–156. PMID:16398064

**21.** Cui Y, Doupe M, Katz A, Nyhof P, Forget EL. Economic evaluation of Manitoba health lines in the management of congestive heart failure. Healthc Policy 2013;9(2):36–50. PMID:24359716

**22.** Dadosky A, Overbeck H, Barbetta L, Bertke K, Corl M, Daly K, Hiles N, Rector N, Chung E, Menon S. Telemanagement of heart failure patients across the post-acute care continuum. Telemed e-Health 2018;24(5):360–366. PMID:28910238

**23.** Dale J, Caramlau I, Docherty A, Sturt J, Hearnshaw H. Telecare motivational interviewing for diabetes patient education and support: a randomised controlled trial based in primary care comparing nurse and peer supporter delivery. Trials 2007;8. PMID:17598895

**24.** Dansky KH, Vasey J, Bowles K. Impact of telehealth on clinical outcomes in patients with heart failure. Clin Nurs Res 2008;17(3):182–199. PMID:18617707

**25.** Dansky K, Vasey J. Managing heart failure patients after formal homecare. Telemed J E Health 2009;15(10):983–991. PMID:19929234

**26.** de la Porte PW, Lok DJA, Van Veldhuisen DJ, Van Wijngaarden J, Cornel JH, Zuirhoff NPA, Badings E, Hoes AW. Added value of a physician-and-nurse-directed heart failure clinic: results from the Deventer-Alkmaar heart failure study. Heart 2007;93(7):819–825. PMID:17065182

**27.** De Simone A, Leoni L, Luzi M, Amellone C, Stabile G, La Rocca V, Capucci A, D’Onofrio A, Ammendola E, Accardi F, Valsecchi S, Buja G. Remote monitoring improves outcome after ICD implantation: the clinical efficacy in the management of heart failure (EFFECT) study. Europace 2015;17(8):1267–1275. PMID:25842271

**28.** de Vries AE, De Jong RM, Van Der Wal MH, Jaarsma T, Van Dijk RB, Hillege HL. The value of INnovative ICT guided disease management combined with Telemonitoring in OUtpatient clinics for Chronic Heart failure patients. Design and methodology of the IN TOUCH study: a multicenter randomised trial. BMC Health Serv Res 2011;11. PMID:21752280

**29.** Dienstl M, Kempf K, Schulz C, Kruse J, Martin S. Effect of telemedicine on glucometabolic control and quality of life in patients with type 2 diabetes mellitus. Diabetol und Stoffwechsel 2011;6(3):164–169. [doi: 10.1055/S-0031-1271460/ID/23]

**30.** Dixon RF, Zisser H, Layne JE, Barleen NA, Miller DP, Moloney DP, Majithia AR, Gabbay RA, Riff J. A virtual type 2 diabetes clinic using continuous glucose monitoring and endocrinology visits. J Diabetes Sci Technol 2020;14(5):908–911. PMID:31762302

**31.** Doyle C, Bhar S, Fearn M, Ames D, Osborne D, You E, Gorelik A, Dunt D. The impact of telephone-delivered cognitive behaviour therapy and befriending on mood disorders in people with chronic obstructive pulmonary disease: A randomized controlled trial. Br J Health Psychol 2017;22(3):542–556. PMID:28544504

**32.** Durso SC, Wendel I, Letzt AM, Lefkowitz J, Kaseman DF, Seifert RF. Older adults using cellular telephones for diabetes management: a pilot study. Medsurg Nurs 2003;12(5):313–317. PMID:14608688

**33.** Egede LE, Walker RJ, Payne EH, Knapp RG, Acierno R, Frueh BC. Effect of psychotherapy for depression via home telehealth on glycemic control in adults with type 2 diabetes: Subgroup analysis of a randomized clinical trial. J Telemed Telecare 2018;24(9):596–602. PMID:28945160

**34.** Egede LE, Williams JS, Voronca DC, Gebregziabher M, Lynch CP. Telephone-delivered behavioral skills intervention for African American adults with type 2 diabetes: a randomized controlled trial. J Gen Intern Med 2017;32(7):775–782. PMID:28337686

**35.** Ell K, Katon W, Lee PJ, Kapetanovic S, Guterman J, Xie B, Chou CP. Depressive symptom deterioration among predominantly Hispanic diabetes patients in safety net care. Psychosomatics 2012;53(4):347–355. PMID:22458987

**36.** Farrero E, Escarrabill J, Prats E, Maderal M, Manresa F. Impact of a hospital-based home-care program on the management of COPD patients receiving long-term oxygen therapy. Chest Chest; 2001;119(2):364–369. PMID:11171710

**37.** Farsaei S, Sabzghabaee AM, Zargarzadeh AH, Amini M. Effect of pharmacist-led patient education on glycemic control of type 2 diabetics: a randomized controlled trial. J Res Med Sci 2011;16(1):43. PMID:21448382

**38.** Gámez-López AL, Bonilla-Palomas JL, Anguita-Sánchez M, Castillo-Domínguez JC, Arizón Del Prado JM, Suárez De Lezo J. [Effects of three different disease management programs on outcomes in patients hospitalized with heart failure: a randomized trial]. Med Clin (Barc) 2012;138(5):192–198. PMID:21605879

**39.** Gellis ZD, Kenaley B, McGinty J, Bardelli E, Davitt J, Ten Have T. Outcomes of a telehealth intervention for homebound older adults with heart or chronic respiratory failure: a randomized controlled trial. Gerontologist Gerontologist; 2012 Aug;52(4):541–552. PMID:22241810

**40.** González-Guerrero JL, Hernández-Mocholi MA, Ribera-Casado JM, García-Mayolín N, Alonso-Fernández T, Gusi N. Cost-effectiveness of a follow-up program for older patients with heart failure: a randomized controlled trial. Eur Geriatr Med 2018;9(4):523–532. PMID:34674493

**41.** Gorodeski EZ, Moennich LA, Riaz H, Jehi L, Young JB, Tang WHW. Virtual versus in-person visits and appointment no-show rates in heart failure care transitions. Circ Hear Fail 2020;13(8):298–300. PMID:32762457

**42.** Hallberg SJ, McKenzie AL, Williams PT, Bhanpuri NH, Peters AL, Campbell WW, Hazbun TL, Volk BM, McCarter JP, Phinney SD, Volek JS. Effectiveness and safety of a novel care model for the management of type 2 diabetes at 1 year: an open-label, non-randomized, controlled study. Diabetes Ther 2018;9(2):583–612. PMID:29417495

**43.** Hansen CR, Perrild H, Koefoed BG, Zander M. Video consultations as add-on to standard care among patients with type 2 diabetes not responding to standard regimens: a randomized controlled trial. Eur J Endocrinol 2017;176(6):727–736. PMID:28325823

**44.** Haynes SC, Tancredi DJ, Tong K, Hoch JS, Ong MK, Ganiats TG, Evangelista LS, Black JT, Auerbach A, Romano PS. Association of adherence to weight telemonitoring with health care use and death: a secondary analysis of a randomized clinical trial. JAMA Netw Open 2020;3(7). PMID:32648924

**45.** Herold R, van den Berg N, Dörr M, Hoffmann W. Telemedical care and monitoring for patients with chronic heart failure has a positive effect on survival. Health Serv Res 2018;53(1):532–555. PMID:28138988

**46.** Holmen H, Wahl A, Torbjørnsen A, Jenum AK, Småstuen MC, Ribu L. Stages of change for physical activity and dietary habits in persons with type 2 diabetes included in a mobile health intervention: the Norwegian study in RENEWING HEALTH. BMJ open diabetes Res care 2016;4(1). PMID:27239317

**47.** Hsu WC, Lau KHK, Huang R, Ghiloni S, Le H, Gilroy S, Abrahamson M, Moore J. Utilization of a cloud-based diabetes management program for insulin initiation and titration enables collaborative decision making between healthcare providers and patients. Diabetes Technol Ther 2016;18(2):59–67. PMID:26645932

**48.** Huizinga MM, Gebretsadik T, Garcia Ulen C, Shintani AK, Michon SR, Shackleford LO, Wolff KL, Brown AW, Rothman RL, Elasy TA. Preventing glycaemic relapse in recently controlled type 2 diabetes patients: a randomised controlled trial. Diabetologia 2010;53(5):832–839. PMID:20084363

**49.** Inoriza JM, Ibañez A, Pérez-Berruezo X, Inoriza-Nadal C, Coderch J. [Effectiveness and economic impact of a program of integrated care with telemedicine support on insulin-treated type 2 diabetic patients (Study GITDIABE)]. Aten Primaria 2016;49(3):131–139. PMID:27423246

**50.** Jakobsen AS, Laursen LC, Rydahl-Hansen S, Østergaard B, Gerds TA, Emme C, Schou L, Phanareth K. Home-based telehealth hospitalization for exacerbation of chronic obstructive pulmonary disease: findings from “the virtual hospital” trial. Telemed J E Health 2015;21(5):364–373. PMID:25654366

**51.** Jakobsson S, Irewall AL, Bjorklund F, Mooe T. Cardiovascular secondary prevention in high-risk patients: a randomized controlled trial sub-study. BMC Cardiovasc Disord 2015;15(1). PMID:26466804

**52.** Jerant AF, Azari R, Martinez C, Nesbitt TS. A randomized trial of telenursing to reduce hospitalization for heart failure: patient-centered outcomes and nursing indicators. Home Health Care Serv Q 2003;22(1):1–20. PMID:12749524

**53.** Jiménez-Marrero S, Yun S, Cainzos-Achirica M, Enjuanes C, Garay A, Farre N, Verdú JM, Linas A, Ruiz P, Hidalgo E, Calero E, Comín-Colet J. Impact of telemedicine on the clinical outcomes and healthcare costs of patients with chronic heart failure and mid-range or preserved ejection fraction managed in a multidisciplinary chronic heart failure programme: A sub-analysis of the iCOR randomized. J Telemed Telecare 2020;26(1–2):64–72. PMID:30193564

**54.** Kashem A, Droogan MT, Santamore WP, Wald JW, Bove AA. Managing heart failure care using an internet-based telemedicine system. J Card Fail 2008;14(2):121–126. PMID:18325458

**55.** Kashem A, Droogan MT, Santamore WP, Wald JW, Marble JF, Cross RC, Bove AA. Web-based Internet telemedicine management of patients with heart failure. Telemed J E Health Telemed J E Health; 2006 Aug;12(4):439–447. PMID:16942416

**56.** Kaur R, Kajal KS, Kaur A, Singh P. Telephonic consultation and follow-up in diabetics: impact on metabolic profile, quality of life, and patient compliance. N Am J Med Sci 2015;7(5):199–207. PMID:26110131

**57.** Kessler R, Casan-Clara P, Koehler D, Tognella S, Viejo JL, Dal Negro RW, Díaz-Lobato S, Reissig K, González-Moro JMR, Devouassoux G, Chavaillon JM, Botrus P, Arnal JM, Ancochea J, Bergeron-Lafaurie A, De Abajo C, Randerath WJ, Bastian A, Cornelissen CG, Nilius G, Texereau JB, Bourbeau J. COMET: a multicomponent home-based disease-management programme versus routine care in severe COPD. Eur Respir J 2018;51(1). PMID:29326333

**58.** King AB, Wolfe GS. Evaluation of a diabetes specialist-guided primary care diabetes treatment program. J Am Acad Nurse Pract 2009;21(1):24-30.

**59.** Kobb R, Hoffman N, Lodge R, Kline S. Enhancing elder chronic care through technology and care coordination: report from a pilot. Telemed J E Health 2003;9(2):189–195. PMID:12855041

**60.** Koehler F, Koehler K, Deckwart O, Prescher S, Wegscheider K, Kirwan BA, Winkler S, Vettorazzi E, Bruch L, Oeff M, Zugck C, Doerr G, Naegele H, Störk S, Butter C, Sechtem U, Angermann C, Gola G, Prondzinsky R, Edelmann F, Spethmann S, Schellong SM, Schulze PC, Bauersachs J, Wellge B, Schoebel C, Tajsic M, Dreger H, Anker SD, Stangl K. Efficacy of telemedical interventional management in patients with heart failure (TIM-HF2): a randomised, controlled, parallel-group, unmasked trial. Lancet (London, England) 2018;392(10152):1047–1057. PMID:30153985

**61.** Koehler F, Winkler S, Schieber M, Sechtem U, Stangl K, Böhm M, Boll H, Baumann G, Honold M, Koehler K, Gelbrich G, Kirwan BA, Anker SD. Impact of remote telemedical management on mortality and hospitalizations in ambulatory patients with chronic heart failure: the telemedical interventional monitoring in heart failure study. Circulation 2011;123(17):1873–1880. PMID:21444883

**62.** Koehler F, Winkler S, Schieber M, Sechtem U, Stangl K, Böhm M, De Brouwer S, Perrin E, Baumann G, Gelbrich G, Boll H, Honold M, Koehler K, Kirwan BA, Anker SD. Telemedicine in heart failure: pre-specified and exploratory subgroup analyses from the TIM-HF trial. Int J Cardiol 2012;161(3):143–150. PMID:21982700

**63.** Krein SL, Klamerus ML, Vijan S, Lee JL, Fitzgerald JT, Pawlow A, Reeves P, Hayward RA. Case management for patients with poorly controlled diabetes: a randomized trial. Am J Med 2004;116(11):732–739. PMID:15144909

**64.** LaFramboise LM, Todero CM, Zimmerman L, Agrawal S. Comparison of Health Buddy with traditional approaches to heart failure management. Fam Community Health 2003;26(4):275–288. PMID:14528134

**65.** Lam A. Practice innovations: delivering medication therapy management services via Videoconference interviews. Consult Pharm 2011;26(10):764–773. PMID:22005142

**66.** Lauffenburger JC, Ghazinouri R, Jan S, Makanji S, Ferro CA, Lewey J, Wittbrodt E, Lee J, Haff N, Fontanet CP, Choudhry NK. Impact of a novel pharmacist-delivered behavioral intervention for patients with poorly-controlled diabetes: The ENhancing outcomes through Goal Assessment and Generating Engagement in Diabetes Mellitus (ENGAGE-DM) pragmatic randomized trial. PLoS One 2019;14(4). PMID:30939143

**67.** Lauffenburger JC, Lewey J, Jan S, Makanji S, Ferro CA, Krumme AA, Lee J, Ghazinouri R, Haff N, Choudhry NK. Effectiveness of targeted insulin-adherence interventions for glycemic control using predictive analytics among patients with type 2 diabetes: a randomized clinical trial. JAMA Netw open JAMA Netw Open; 2019 Mar 1;2(3):e190657. PMID:30874782

**68.** Layman SN, Elliott W V., Regen SM, Keough LA. Implementation of a pharmacist-led transitional care clinic. Am J Health Syst Pharm 2020;77(12):966–971. PMID:32374382

**69.** Lehmann CA, Mintz N, Giacini JM. Impact of telehealth on healthcare utilization by congestive heart failure patients. Disease Management and Health Outcomes. 2006;14(3):163-169.

**70.** Leichter SB, Bowman K, Adkins RA, Jelsovsky Z. Impact of remote management of diabetes via computer: the 360 study--a proof-of-concept randomized trial. Diabetes Technol Ther 2013;15(5):434–438. PMID:23537419

**71.** Lilholt PH, Udsen FW, Ehlers L, Hejlesen OK. Telehealthcare for patients suffering from chronic obstructive pulmonary disease: effects on health-related quality of life: results from the Danish “TeleCare North” cluster-randomised trial. BMJ Open 2017;7(5). PMID:28490555

**72.** Liou JK, Soon MS, Chen CH, Huang TF, Chen YP, Yeh YP, Chang CJ, Kuo SJ, Hsieh MC. Shared care combined with telecare improves glycemic control of diabetic patients in a rural underserved community. Telemed J E Health 2014;20(2):175–178. PMID:24320193

**73.** Litke J, Spoutz L, Ahlstrom D, Perdew C, Llamas W, Erickson K. Impact of the clinical pharmacy specialist in telehealth primary care. Am J Health Syst Pharm 2018;75(13):982–986.

**74.** López Cabezas C, Falces Salvador C, Cubí Quadrada D, Arnau Bartés A, Ylla Boré M, Muro Perea N, Homs Peipoch E. Randomized clinical trial of a postdischarge pharmaceutical care program vs regular follow-up in patients with heart failure. Farm Hosp Farm Hosp; 2006;30(6):328–342. PMID:17298190

**75.** Lyons I, Barber N, Raynor DK, Wei L. The Medicines Advice Service Evaluation (MASE): a randomised controlled trial of a pharmacist-led telephone based intervention designed to improve medication adherence. BMJ Qual Saf 2016;25(10):759–769. PMID:26755665

**76.** Majithia AR, Kusiak CM, Lee AA, Colangelo FR, Romanelli RJ, Robertson S, Miller DP, Erani DM, Layne JE, Dixon RF, Zisser H. Glycemic outcomes in adults with type 2 diabetes participating in a continuous glucose monitor-driven virtual diabetes clinic: prospective trial. J Med Internet Res 2020;22(8). PMID:32856597

**77.** Martinez AS, Saef J, Paszczuk A, Bhatt-Chugani H. Implementation of a pharmacist-managed heart failure medication titration clinic. Am J Health Syst Pharm 2013;70(12):1070–1076. PMID:23719886

**78.** Mayes PA, Silvers A, Prendergast JJ. New direction for enhancing quality in diabetes care: utilizing telecommunications and paraprofessional outreach workers backed by an expert medical team. Telemed J E Health 2010;16(3):358–363. PMID:20406123

**79.** McElroy I, Sareh S, Zhu A, Miranda G, Wu H, Nguyen M, Shemin R, Benharash P. Use of digital health kits to reduce readmission after cardiac surgery. J Surg Res 2016;204(1):1–7. PMID:27451860

**80.** Moayeri F, Dunt D, Hsueh YS (Arthur), Doyle C. Cost-utility analysis of telephone-based cognitive behavior therapy in chronic obstructive pulmonary disease (COPD) patients with anxiety and depression comorbidities: an application for willingness to accept concept. Expert Rev Pharmacoecon Outcomes Res 2019;19(3):331–340. PMID:30324818

**81.** Moore AB, Krupp JE, Dufour AB, Sircar M, Travison TG, Abrams A, Farris G, Mattison MLP, Lipsitz LA. Improving transitions to postacute care for elderly patients using a novel video-conferencing program: ECHO-Care Transitions. Am J Med 2017;130(10):1199–1204. PMID:28551043

**82.** Morguet AJ, Kühnelt P, Kallel A, Jaster M, Schultheiss HP. Impact of telemedical care and monitoring on morbidity in mild to moderate chronic heart failure. Cardiology 2008;111(2):134–139. PMID:18376125

**83.** Mortara A, Pinna GD, Johnson P, Maestri R, Capomolla S, La Rovere MT, Ponikowski P, Tavazzi L, Sleight P. Home telemonitoring in heart failure patients: the HHH study (Home or Hospital in Heart Failure). Eur J Heart Fail 2009;11(3):312–318. PMID:19228800

**84.** Moyer-Knox D, Mueller TM, Vuckovic K, Mischke L, Williams RE. Remote titration of carvedilol for heart failure patients by advanced practice nurses. J Card Fail 2004;10(3):219–224. PMID:15190531

**85.** Myers A, Presswala L, Bissoonauth A, Gulati N, Zhang M, Izard S, Kozikowski A, Meyers K, Pekmezaris R. Telemedicine for disparity patients with diabetes: the feasibility of utilizing telehealth in the management of uncontrolled type 2 diabetes in Black and Hispanic disparity patients; a pilot study. J Diabetes Sci Technol 2020;15(5):1034–1041. PMID:32865027

**86.** Nakayama A, Takayama N, Kobayashi M, Hyodo K, Maeshima N, Takayuki F, Morita H, Komuro I. Remote cardiac rehabilitation is a good alternative of outpatient cardiac rehabilitation in the COVID-19 era. Environ Health Prev Med 2020;25(1). PMID:32891113

**87.** Nguyen HQ, Donesky-Cuenco DA, Wolpin S, Reinke LF, Benditt JO, Paul SM, Carrieri-Kohlman V. Randomized controlled trial of an internet-based versus face-to-face dyspnea self-management program for patients with chronic obstructive pulmonary disease: pilot study. J Med Internet Res 2008;10(2). PMID:18417444

**88.** Nield M, Hoo GWS. Real-time telehealth for COPD self-management using Skype. COPD J Chronic Obstr Pulm Dis 2012;9(6):611–619. PMID:22946768

**89.** Nouryan CN, Morahan S, Pecinka K, Akerman M, Lesser M, Chaikin D, Castillo S, Zhang M, Pekmezaris R. Home telemonitoring of community-dwelling heart failure patients after home care discharge. Telemed e-Health 2019;25(6):447–454. PMID:30036166

**90.** Odegard PS, Goo A, Hummel J, Williams KL, Gray SL. Caring for poorly controlled diabetes mellitus: a rando

**91.** Odeh B, Kayyali R, Nabhani-Gebara S, Philip N, Robinson P, Wallace CR. Evaluation of a Telehealth Service for COPD and HF patients: Clinical outcome and patients’ perceptions. J Telemed Telecare 2015;21(5):292–297. PMID:25766852.

**92.** Oh JA, Kim HS, Yoon KH, Choi ES. A telephone-delivered intervention to improve glycemic control in type 2 diabetic patients. Yonsei Med J 2003;44(1):1–8. PMID:12619168

**93.** Paré G, Sicotte C, St.-Jules D, Gauthier R. Cost-minimization analysis of a telehomecare program for patients with chronic obstructive pulmonary disease. Telemed J E Health 2006;12(2):114–121. PMID:16620165

**94.** Pedone C, Rossi FF, Cecere A, Costanzo L, Antonelli Incalzi R. Efficacy of a physician-led multiparametric telemonitoring system in very old adults with heart failure. J Am Geriatr Soc 2015;63(6):1175–1180. PMID:26031737

**95.** Pérez-Rodríguez G, Brito-Zurita OR, Sistos-Navarro E, Benítez-Aréchiga ZM, Sarmiento-Salazar GL, Vargas-Lizárraga JF. [Telemetric monitoring reduces visits to the emergency room and cost of care in patients with chronic heart failure]. Cir Cir 2015;83(4):279–285. PMID:26116037

**96.** Polonsky WH, Layne JE, Parkin CG, Kusiak CM, Barleen NA, Miller DP, Zisser H, Dixon RF. Impact of participation in a virtual diabetes clinic on diabetes-related distress in individuals with type 2 diabetes. Clin Diabetes 2020;38(4):357–362. PMID:33132505

**97.** Quinn CC, Shardell MD, Terrin ML, Barr EA, Park D, Shaikh F, Guralnik JM, Gruber-Baldini AL. Mobile diabetes intervention for glycemic control in 45- to 64-year-old persons with type 2 diabetes. J Appl Gerontol 2016;35(2):227–243. PMID:25098253

**98.** Ringbæk T, Green A, Laursen LC, Frausing E, Brøndum E, Ulrik CS. Effect of tele health care on exacerbations and hospital admissions in patients with chronic obstructive pulmonary disease: a randomized clinical trial. Int J Chron Obstruct Pulmon Dis 2015;10(1):1801–1808. PMID:26366072

**99.** Rodríguez-Idígoras MI, Sepúlveda-Muñoz J, Sánchez-Garrido-Escudero R, Martínez-González JL, Escolar-Castelló JL, Paniagua-Gómez IM, Bernal-López R, Fuentes-Simón M V., Garófano-Serrano D. Telemedicine influence on the follow-up of type 2 diabetes patients. Diabetes Technol Ther 2009;11(7):431–437. PMID:19580356

**100.** Rüter G, Mons U, Brenner H. New approaches to optimize general practitioner care in type 2 diabetes. Results of the DIANA study. Diabetologe. 2014;10(3):207-216.

**101.** Salvo MC, Brooks AMC. Glycemic control and preventive care measures of indigent diabetes patients within a pharmacist-managed insulin titration program vs standard care. Ann Pharmacother 2012;46(1):29–34. PMID:22202497

**102.** Sarayani A, Mashayekhi M, Nosrati M, Jahangard-Rafsanjani Z, Javadi M, Saadat N, Najafi S, Gholami K. Efficacy of a telephone-based intervention among patients with type-2 diabetes; a randomized controlled trial in pharmacy practice. Int J Clin Pharm 2018;40(2):345–353. PMID:29435911

**103.** Scalvini S, Capomolla S, Zanelli E, Benigno M, Domenighini D, Paletta L, Glisenti F, Giordano A. Effect of home-based telecardiology on chronic heart failure: costs and outcomes. J Telemed Telecare 2005;11 Suppl 1(SUPPL. 1):16–18. PMID:16035980

**104.** Scalvini S, Zanelli E, Paletta L, Benigno M, Domeneghini D, de Giuli F, Giordano A, Glisenti F. Chronic heart failure home-based management with a telecardiology system: a comparison between patients followed by general practitioners and by a cardiology department. J Telemed Telecare 2006;12 Suppl 1(SUPPL. 1). PMID:16884578

**105.** Schmidt K, Caudill JA, Hamilton T. Impact of clinical pharmacy specialists on glycemic control in veterans with type 2 diabetes. Am J Health Syst Pharm 2019;76(Supplement_1):S28–S33. PMID:30753315

**106.** Smith B, Hughes-Cromwick PF, Forkner E, Galbreath AD. Cost-effectiveness of telephonic disease management in heart failure. Am J Manag Care 2008;14(2):106–115. PMID:18269306

**107.** Sorocco KH, Bratkovich KL, Wingo R, Qureshi SM, Mason PJ. Integrating care coordination home telehealth and home based primary care in rural Oklahoma: a pilot study. Psychol Serv 2013;10(3):350–352. PMID:23937085

**108.** Steventon A, Bardsley M, Doll H, Tuckey E, Newman SP. Effect of telehealth on glycaemic control: analysis of patients with type 2 diabetes in the Whole Systems Demonstrator cluster randomised trial. BMC Health Serv Res 2014;14(1). PMID:25100190

**109.** Stewart SA, Worth L, Burton C. Evaluating a Telehealth Follow-up Program for Cardiology Patients Using Administrative Data. *Stud Health Technol Inform.* 2015;209:156-161. PMID: 25980719

**110.** Stone RA, Rao RH, Sevick MA, Cheng C, Hough LJ, Macpherson DS, Franko CM, Anglin RA, Obrosky DS, DeRubertis FR. Active Care Management Supported by Home Telemonitoring in Veterans With Type 2 DiabetesThe DiaTel randomized controlled trial. Diabetes Care 2010;33(3):478–484. PMID:20009091

**111.** Tabak M, Brusse-Keizer M, van der Valk P, Hermens H, Vollenbroek-Hutten M. A telehealth program for self-management of COPD exacerbations and promotion of an active lifestyle: a pilot randomized controlled trial. Int J Chron Obstruct Pulmon Dis 2014;9:935. PMID:25246781

**112.** Taylor S, Hourihan F, Krass I, Armour C. Measuring consumer preference for models of diabetes care delivered by pharmacists. Pharm Pract (Granada) 2009;7(4):195-204.

**113.** Veenstra W, op den Buijs J, Pauws S, Westerterp M, Nagelsmit M. Clinical effects of an optimised care program with telehealth in heart failure patients in a community hospital in the Netherlands. Neth Heart J 2015;23(6):334–340. PMID:25947078

**114.** Vidula H, Cheyne C, Dick S, et al. Feasibility and Patient Perceptions of Tele-Videoconferencing Visits for LVAD Patients (TeleLVAD Study). The Journal of heart and lung transplantation: the official publication of the International Society for Heart Transplantation 2020;39(4):S133.

**115.** Vitacca M, Bianchi L, Guerra A, Fracchia C, Spanevello A, Balbi B, Scalvini S. Tele-assistance in chronic respiratory failure patients: a randomised clinical trial. Eur Respir J 2009;33(2):411–418. PMID:18799512

**116.** Wakefield BJ, Holman JE, Ray A, Scherubel M, Adams MR, Hills SL, Rosenthal GE. Outcomes of a home telehealth intervention for patients with diabetes and hypertension. Telemed J E Health 2012;18(8):575–579. PMID:22873700

**117.** Wakefield BJ, Ward MM, Holman JE, Ray A, Scherubel M, Burns TL, Kienzle MG, Rosenthal GE. Evaluation of home telehealth following hospitalization for heart failure: a randomized trial. Telemed J E Health 2008;14(8):753–761. PMID:18954244

**118.** Whitten P, Mickus M. Home telecare for COPD/CHF patients: outcomes and perceptions. J Telemed Telecare 2007;13(2):69–73. PMID:17359569

**119.** Wild SH, Hanley J, Lewis SC, McKnight JA, McCloughan LB, Padfield PL, Parker RA, Paterson M, Pinnock H, Sheikh A, McKinstry B. Supported telemonitoring and glycemic control in people with type 2 diabetes: the telescot diabetes pragmatic multicenter randomized controlled trial. PLoS Med 2016;13(7). PMID:27458809

**120.** Woodend AK, Sherrard H, Fraser M, Stuewe L, Cheung T, Struthers C. Telehome monitoring in patients with cardiac disease who are at high risk of readmission. Heart Lung 2008;37(1):36–45. PMID:18206525

**121.** Wright EA, Graham JH, Maeng D, Tusing L, Zaleski L, Martin R, Seipp R, Citsay B, McDonald B, Bolesta K, Chaundy K, Medico CJ, Gunderman S, Leri F, Guza K, Price R, Gregor C, Parry DT. Reductions in 30-day readmission, mortality, and costs with inpatient-to-community pharmacist follow-up. J Am Pharm Assoc (2003) 2019;59(2):178–186. PMID:30655090

**122.** Wu RC, Delgado D, Costigan J, MacIver J, Ross H. Pilot study of an Internet patient-physician communication tool for heart failure disease management. J Med Internet Res 2005;7(1). PMID:15829480

**123.** Yan Y, Liu L, Zeng J, Zhang L. Evaluation and exploration on the effect of the management of chronic obstructive pulmonary disease in rural areas through an Internet-based network consulting room. Med Princ Pract 2018;27(3):222–226. PMID:29558756

**124.** Yoo HJ, Park MS, Kim TN, Yang SJ, Cho GJ, Hwang TG, Baik SH, Choi DS, Park GH, Choi KM. A ubiquitous chronic disease care system using cellular phones and the internet. Diabet Med 2009;26(6):628–635. PMID:19538239
