# Supplementary material for: Telehealth for the Longitudinal Management of Chronic Conditions: Systematic Review
Source: J Med Internet Res. 2022 Aug 26;24(8):e37100. doi: 10.2196/37100 (PMC9463619; doi:10.2196/37100)
Supplement: Multimedia Appendix 6 [file jmir_v24i8e37100_app6.docx]

**Multimedia Appendix 6:** Ad Horizon Scan to Identify Relevant in Progress Studies

| First author, year | Recruitment target  Study design | Disease state | Intervention/Comparator | Planned duration | Clinical trials number |
| --- | --- | --- | --- | --- | --- |
| **Rovner, 2018 {Rovner, 2018 #25851}** | African Americans,  >50 years,  T1DM^a^ or T2DM^b^, after  DM^c^-related emergency department visit  Single blind, randomized controlled trial | T2DM | Multi-component intervention including behavioral activation and the facilitation of telehealth visits with primary care and a DM nurse educator vs multi-component intervention without telehealth facilitation or behavioral activation | 12 months | [NCT03466866](https://clinicaltrials.gov/show/NCT03466866) |
| **Rodrigues, 2019 {Rodrigues, 2019 #25852}** | Patients with T2DM, >18 years, referred from primary care  Pragmatic, open-label, phase 2, non-inferiority, randomized controlled trial | T2DM | Teleconsultation with endocrinologist by video vs face-to-face | Unclear | WHO^d^ Clinical Trials Registry ID: RBR-8gpgyd |
| **Komkov, date unknown {Denis Komkov, #25853}** | Patients with CHF^e^ discharged from the hospital  Randomized controlled trial | CHF | Short-term education and active telephone calls by physician vs usual care | 12 months | Unknown |

^a^T1DM = type 1 diabetes mellitus

^b^T2DM: type 2 diabetes mellitus

^c^DM: diabetes mellitus

^d^WHO: World Health Organization

^e^CHF: congestive heart failure
